# Correction: Migraine and risk of premature myocardial infarction and stroke among men and women: A Danish population-based cohort study

**DOI:** 10.1371/journal.pmed.1004353

**Published:** 2024-02-13

**Authors:** Cecilia Hvitfeldt Fuglsang, Lars Pedersen, Morten Schmidt, Jan P. Vandenbroucke, Hans Erik Bøtker, Henrik Toft Sørensen

The first author’s name is indexed incorrectly. The correct citation is: Fuglsang CH, Pedersen L, Schmidt M, Vandenbroucke JP, Bøtker HE, Sørensen HT (2023) Migraine and risk of premature myocardial infarction and stroke among men and women: A Danish population-based cohort study. PLoS Med 20(6): e1004238. https://doi.org/10.1371/journal.pmed.1004238
