# Supplementary material for: Migraine and risk of premature myocardial infarction and stroke among men and women: A Danish population-based cohort study
Source: PLoS Med. 2023 Jun 13;20(6):e1004238. doi: 10.1371/journal.pmed.1004238 (PMC10263301; doi:10.1371/journal.pmed.1004238)
Supplement: S1 Fig — Legend: *Earliest of cardiovascular event, 61st birthday, emigration, death, or administrative censoring (31 December 2018). Based on template by Schneeweiss and colleagues [14]. (DOCX) [file pmed.1004238.s002.docx]

### S1 Fig. Graphical depiction of study design and windows for exclusion, exposure, covariate, and follow-up assessment.


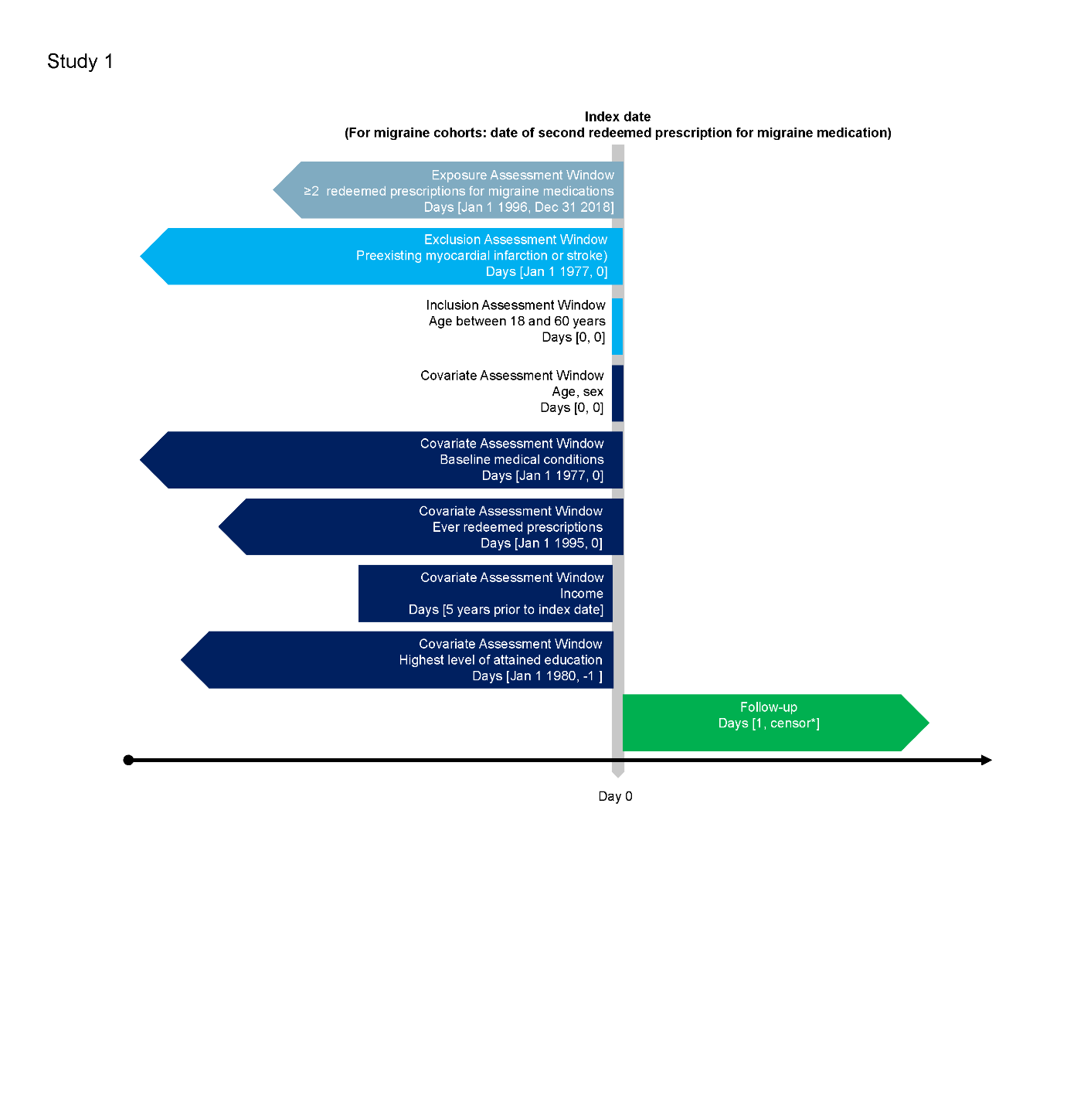


*Earliest of cardiovascular event, 61st birthday, emigration, death, or administrative censoring (31 December 2018). Based on template by Schneeweiss et al. [1]

References

1. Schneeweiss S, Rassen JA, Brown JS, Rothman KJ, Happe L, Arlett P, et al. Graphical Depiction of Longitudinal Study Designs in Health Care Databases. Ann Intern Med. 2019;170(6):398-406. Epub 2019/03/12. doi: 10.7326/m18-3079. PubMed PMID: 30856654.
