# Supplementary material for: Migraine and risk of premature myocardial infarction and stroke among men and women: A Danish population-based cohort study
Source: PLoS Med. 2023 Jun 13;20(6):e1004238. doi: 10.1371/journal.pmed.1004238 (PMC10263301; doi:10.1371/journal.pmed.1004238)
Supplement: S1 Text — (DOCX) [file pmed.1004238.s003.docx]

# Migraine and risk of premature myocardial infarction and stroke among men and women: A Danish Population-based cohort study. Statistical analysis plan

## Aim

To examine the impact of migraine on the association between sex and risk of premature stroke and myocardial infarction

*March 2023:*

*We originally intended this to be a study of interaction between migraine and sex on risk of premature myocardial infarction and stroke. However, it did not seem obvious who were going to be the exposed sex and therefore we changed the study aim to be one of effect modification, i.e. what was the impact of migraine among men and among women. The title was changed accordingly.*

## METHODS

## Setting and design

We will perform a population-based cohort study in Denmark using data obtained from national registries. Individual level data will be linked using the 10-digit civil registration number issued by the Civil Registration system. The source population will be all Danish residents during the period of 1 January 1996 through 31 December 2016.

## Data Sources

The Civil Registration System, CRS

The Danish National Patient Registry, DNPR

The Danish National Prescription Registry, NPR

The Danish Registry of Causes of Death, DRCD

The Danish Integrated Database for Labor Market Research, IDA

The Psychiatric Central Registry, PCR

The Danish Stroke Registry, DSR

## Study cohorts

**MIGRAINE ICD DIAGNOSIS study**

**Migraine cohort:** All patients aged 18-60 with a first-time primary or secondary diagnosis of migraine (inpatient, outpatient, and emergency room diagnoses) recorded in the DNPR during the study period. Index date will be date of diagnosis (admission date). Patients with a diagnosis of stroke or MI (inpatient/outpatient only) recorded in the DNPR before index date or with a diagnosis of migraine prior to the study period will be excluded.

**General population comparison cohort:** From the CRS we will randomly sample 10 comparison cohort members for each migraine patient. Comparison cohort members will be matched on birth year and year of index date. Matching will be done with replacement. They will be assigned the index date corresponding to that of their matched migraine patient. Individuals are eligible for the comparison cohort if they are alive on the index date, have no previous diagnosis of stroke, MI or migraine before the index date.

If a member of the comparison cohort receive a diagnosis of migraine during follow-up, the individual joins the migraine cohort.

**PRESCRIPTIONS study**

**Migraine prescription cohort:** All patients aged 18-60 with at least two redeemed prescriptions for migraine specific medication recorded in the NPR (available from 1995) during the study period. Index date will be date of redemption of second prescription. Patients with a diagnosis of stroke or MI (in/outpatient) recorded in the DNPR before index date and patients with two prescriptions before the study period will be excluded.

**General population comparison cohort:** From the CRS we will randomly sample 10 comparison cohort members for each migraine patient. Comparison cohort members will be matched on birth year and year of index date. Matching will be done with replacement. They will be assigned the index date corresponding to that of their matched migraine patient. Individuals are eligible for the comparison cohort if they are alive on the index date, have no previous diagnosis of stroke, MI, and have not redeemed 2 or more prescriptions of migraine specific medication before the index date.

If a member of the comparison cohort receives two prescriptions of migraine specific medications during follow-up, the individual joins the migraine cohort.

*March 2023:*

*We initially intended to use the migraine cohorts identified by diagnostic codes in the DNPR as the main analysis. However, seeing, as there were relatively few in this cohort we decided to use this as a sensitivity analysis instead.*

## Exposure 1

Gender

## Exposure 2

Migraine

## Outcome

The primary outcome will be occurrence of premature stroke or MI (IN/OUT, A/B diagnoses) (i.e. before the age of 61) recorded in the DNPR.

*March 2023:*

*To increase the validity of the outcome diagnoses we decided to only include inpatient codes. Furthermore, we decided to do separate analyses for ischemic stroke and hemorrhagic stroke.*

## Covariables

Charlson comorbidity index (excluding MI and cerebrovascular disease) (A/B diagnoses, IN/OUT/ED)

CCI score (low, medium, high) (excluding MI and cerebrovascular disease)

Other comorbidities (see Table 1) (A/B diagnoses, IN/OUT/ED)

Socioeconomic status (highest level of education, income and employment status)

Tables of definitions of covariables can be found in the appendix table A2.

*March 2023:*

*Data regarding employment status were incomplete from 2014 and forth, and therefore we did not include this information. We included ever-redeemed prescriptions to the descriptive tables.*

## Statistical analysis

1. We will describe our study-population according to age at index date, aura-status of migraine (for those diagnosed using DNPR), calendar period, highest level of education, and comorbidities (Table 1).
2. We will follow migraine patients and controls from index date until date of stroke or MI, death, emigration, date of 61^th^ birthday, or end of study (31 December 2016), whichever comes first. We will calculate cumulative incidence proportions for the migraine and the control cohort stratified on gender, treating death as a competing risk. We will use Cox regression to calculate crude and adjusted hazard ratios as a measure of relative risk comparing women and men with and without migraine. Adjustments will be made for socioeconomic status and comorbidities. To asses interaction between gender and migraine we will calculate the interaction contrast as well as the attributable fraction resulting from interaction and the relative excess risk due to interaction (RERI) (Table 2). Interaction measurements will be based on standardized incidence rates where women without migraine will be the reference group. We will standardize according to calendar period (1996-2000, 2000-2005, 2006-2010, 2011-2016), age group (18-25, 26-30, 31-35, 36-40, 41-45, 46-50, 51-55, 56-61), and Charlson Comorbidity Index score ( low, medium, high).

## Tables

| **Table 1**  **Baseline characteristics according to gender and migraine status** | | | | |
| --- | --- | --- | --- | --- |
|  | **Women** | | **Men** | |
|  | **No migraine**  **n (%)** | **Migraine**  **n (%)** | **No migraine**  **n (%)** | **Migraine**  **n (%)** |
| **All** |  |  |  |  |
| **Aura** | | | | |
| Yes | - |  | - |  |
| No | - |  | - |  |
| Unknown | - |  | - |  |
| Presciption-based |  |  |  |  |
| **Age, median (IQR)** |  |  |  |  |
| **Calendar period at index date** | | | | |
| 1978-1987 |  |  |  |  |
| 1988-1997 |  |  |  |  |
| 1998-2007 |  |  |  |  |
| 2008-2018 |  |  |  |  |
| **Highest level of education** | | | | |
| Low |  |  |  |  |
| Medium |  |  |  |  |
| High |  |  |  |  |
| Unknown |  |  |  |  |
| **Charlson Comorbidity Index** | | | | |
| Low |  |  |  |  |
| Medium |  |  |  |  |
| High |  |  |  |  |
| **Individual Charlson Comorbidities** | | | | |
| Heart failure |  |  |  |  |
| Peripheral vascular disease |  |  |  |  |
| Dementia |  |  |  |  |
| Chronic pulmonary disease |  |  |  |  |
| Connective tissue disease |  |  |  |  |
| Ulcer disease |  |  |  |  |
| Mild liver disease |  |  |  |  |
| Diabetes |  |  |  |  |
| Hemiplegia |  |  |  |  |
| Moderate to severe renal disease |  |  |  |  |
| Diabetes with end organ failure |  |  |  |  |
| Tumor |  |  |  |  |
| Leukemia |  |  |  |  |
| Lymphoma |  |  |  |  |
| Moderate to severe liver disease |  |  |  |  |
| Metastatic solid tumor |  |  |  |  |
| AIDS |  |  |  |  |
| **Other comorbidities** | | | | |
| Hypertension |  |  |  |  |
| Atrial fibrillation or flutter |  |  |  |  |
| Hyperlipidemia |  |  |  |  |
| Atherosclerosis |  |  |  |  |
| Pulmonary embolism |  |  |  |  |
| Deep vein thrombosis |  |  |  |  |
| Valvular heart disease |  |  |  |  |
| Obesity |  |  |  |  |
| Thyroid disease |  |  |  |  |
| Other alcoholism related disease |  |  |  |  |
| Psychiatric disease |  |  |  |  |
|  |  |  |  |  |

| **Table 2**  **Cumulative incidence and hazard ratios of myocardial infarction (MI) according to gender and migraine-status** | | | | | | | | | |
| --- | --- | --- | --- | --- | --- | --- | --- | --- | --- |
| **Gender** | **Migraine-status** | **Total**  **N** | **Stroke or MI**  **N** | **Peron years**  **Y** | **Cumulative incidence**  **(95% CI)** | **Adjusted HR***  **(95% CI)** | **Interaction** | | |
|  |  |  |  |  |  |  | **Interaction contrast** | **Attributable fraction** | **Relative excess risk due to interaction** |
| All | No migraine |  |  |  |  | Reference | - | - | - |
| All | Migraine |  |  |  |  |  | - | - | - |
| Women | No migraine |  |  |  |  | Reference | Reference | Reference | Reference |
| Women | Migraine |  |  |  |  |  |  |  |  |
| Men | No migraine |  |  |  |  | Reference |  |  |  |
| Men | Migraine |  |  |  |  |  |  |  |  |
| *Adjusted for socioeconomic status and comorbidities | | | | | | | | | |

## Appendix

| **Table A1: Definition of exposures and outcome** | | | | | | | |
| --- | --- | --- | --- | --- | --- | --- | --- |
| **Role in the analysis** | **Variable** | **Data sources** | **Categories** | **ICD8-codes** | **ICD10-codes** | **ATC-codes** | **Notes** |
| **Exposure 1** | **Gender** | CRS | Male  Female |  |  |  |  |
| **Exposure 2** | **Migraine** | DNPR  NPR |  | 346.00  346.08  346.09 | G43.0-43.3  G43.8  G43.9 | N02CC, N02CA01-02,  N02CA04, N02CA52  N02CX01-02 | At least two redemptions.  N02CX01-02 are prophylaxis |
|  | Migraine with aura | DNPR |  | - | G43.1 |  |  |
|  | Migraine without aura | DNPR |  | 346.00  346.08  346.09 | G43.0 |  |  |
| **Outcome** | **Stroke** | DNPR |  | 430, 431,433, 434 | I60, I61,  I63, I64 |  |  |
|  | Ischemic stroke | DNPR |  | 433-434 | I63 |  |  |
|  | Subarachnoid hemorrhage | DNPR |  | 430 | I60 |  |  |
|  | Intracerebral hemorrhage | DNPR |  | 431 | I61 |  |  |
|  | Not specified | DNPR |  | - | I64 |  |  |
|  | **Myocardial infarction** | DNPR |  | 410 | I21 |  |  |
|  | STEMI | DNPR |  |  | I21.0-I21.3 |  |  |
|  | Non-STEMI | DNPR |  |  | I21.4 |  |  |
|  | Unspecified | DNPR |  |  | I21.9 |  |  |

| **Table A2: Definitions of covariables** | | | | | | |
| --- | --- | --- | --- | --- | --- | --- |
| **Variable** | **Data sources** | **Categories** | **ICD8-codes** | **ICD10-codes** | **ATC-codes** | **Notes** |
| Birth year (matched on) | CRS |  |  |  |  |  |
| Socioeconomic status  Highest level of education | IDA | Low (primary or lower secondary)  Medium (upper secondary or academic profession degree)  High (University education at bachelor degrees or higher) or missing |  |  |  |  |
| Socioeconomic status  Income | IDA | Low, medium, high, very high, missing |  |  |  | Based on average of 5 previous years of income. Categories according to percentiles from year previous to index date. If one of income is missing based on average of 4 other years |
| Socioeconomic status  Employment | IDA | Employed, unemployed, early retirement, state pension, under education or missing |  |  |  | Year closest to index year within 5 years up to index year |
| **Charlson comorbidity Index** |  |  |  |  |  |  |
| Heart failure | DNPR |  | 427.09; 427.10; 427.11; 427.19; 428.99; 782.49 | I50; I11.0; I13.0; I13.2 |  | Score: 1 |
| Peripheral vascular disease | DNPR |  | 440; 441; 442; 443; 444; 445 | I70; I71; I72; I73; I74; I77 |  | Score: 1 |
| Dementia | DNPR |  | 290.09-290.19; 293.09 | F00-F03; F05.1; G30 |  | Score: 1 |
| Chronic pulmonary disease | DNPR |  | 490-493; 515-518 | J40-J47; J60-J67; J68.4; J70.1;  J70.3; J84.1; J92.0; J96.1; J98.2; J98.3 |  | Score: 1 |
| Connective tissue disease | DNPR |  | 712; 716; 734; 446; 135.99 | M05; M06; M08; M09;M30;M31;  M32; M33; M34; M35; M36; D86 |  | Score: 1 |
| Ulcer disease | DNPR |  | 530.91; 530.98; 531-534 | K22.1; K25-K28 |  | Score: 1 |
| Mild liver disease | DNPR |  | 571; 573.01; 573.04 | B18; K70.0-K70.3; K70.9; K71; K73; K74; K76.0 |  | Score: 1 |
| Diabetes | DNPR |  | 249.00; 249.06; 249.07; 249.09  250.00; 250.06; 250.07; 250.09 | E10.0, E10.1; E10.9  E11.0; E11.1; E11.9 |  | Score: 1 |
| Hemiplegia | DNPR |  | 344 | G81; G82 |  | Score: 2 |
| Moderate to severe renal disease | DNPR |  | 403; 404; 580-583; 584; 590.09; 593.19; 753.10-753.19; 792 | I12; I13; N00-N05; N07; N11; N14; N17-N19; Q61 |  | Score: 2 |
| Diabetes with end organ damage | DNPR |  | 249.01-249.05; 249.08  250.01-250.05; 250.08 | E10.2-E10.8  E11.2-E11.8 |  | Score: 2 |
| Any tumor | DNPR |  | 140-194 | C00-C75 |  | Score: 2 |
| Leukemia | DNPR |  | 204-207 | C91-C95 |  | Score: 2 |
| Lymphoma | DNPR |  | 200-203; 275.59 | C81-C85; C88; C90; C96 |  | Score: 2 |
| Moderate to severe liver disease | DNPR |  | 070.00; 070.02; 070.04; 070.06; 070.08; 573.00; 456.00-456.09 | B15.0; B16.0; B16.2; B19.0; K70.4; K72; K76.6; I85 |  | Score: 3 |
| Metastatic solid tumor | DNPR |  | 195-198; 199 | C76-C80 |  | Score: 6 |
| AIDS | DNPR |  | 079.83 | B21-B24 |  | Score: 6 |
| **Other comorbidities** |  |  |  |  |  |  |
| Hypertension | DNPR  NPR |  | 400-404.99 | I10-I15 | C02A, C02B, C02C, C02DA, C02L, C03A, C03B, C03D, C03E, C03X, C07C, C07D, C08G, C09BA, C09DA, C09XA52, C02DB, C02DD, C02DG, C04, C05, C07, C07F, C08, C09BB, C09DB, C09 | Combination treatment of at least two redeemed prescriptions for different types of drugs within 180 days prior to index date |
| Atrial fibrillation or flutter | DNPR |  | 427.93  427.94 | I48 |  |  |
| Hyperlipidemia | DNPR |  | 272.00, 272.01, 272.08, 272.09 | E78.0-E78.5 |  |  |
| Atherosclerosis | DNPR |  | 440.09, 440.19, 440.20, 440.21, 440.28, 440.29, 440.30, 440.39, 440.99 | I70.0-I70.09 |  |  |
| Pulmonary embolism | DNPR |  | 450.99 | I26 |  |  |
| Deep vein thrombosis | DNPR |  | 451.00 | I80.1-I80.3 |  |  |
| Valvular heart disease | DNPR |  | 394, 395 | I05, I06, I34, I35, I39, I39.1, I51.1A |  |  |
| Obesity | DNPR |  | 277 | E65-E68 |  |  |
| Thyroid disease | DNPR |  | 240-246 | E00-E07 |  |  |
| Other alcoholism related disease | DNPR  NPR |  | 291, 303, 979, 980, 577.10, 571.09, 571.10 | F10 (except F10.0), Z72.1, K86.0, K70, G31.2, G62.1, G72.1, I42.6, K29.2, Z71.4 | Before 31 December 2000: V03AA  After 1 January 2001:  N07BB |  |
| Mood disorder | DNPR |  | 296.x9 (excluding 296.89), 298.09, 298.19, 300.49, 301.19 | F30-F39 |  |  |
| COPD | DNPR |  | 490, 491, 492 | J40, J41, J42, J43, J44 |  |  |
| **Prescriptions** |  |  |  |  |  |  |
| Beta-blockers | NPR |  |  |  | C07 |  |
| ACE-inhibitor/ATII-receptor blocker | NPR |  |  |  | C09A, C09B, C09C, C09D |  |
| Diuretics | NPR |  |  |  | C03 |  |
| Lipid-lowering drugs | NPR |  |  |  | C10 |  |
| Vitamin K antagonists | NPR |  |  |  | B01AA |  |
| NOAC | NPR |  |  |  | B01AE, B01AF |  |
| Clopidogrel | NPR |  |  |  | B01AC04 |  |
| Platelet aggregation inhibitors, excl. clopidogrel and acetylsalicylic acid | NPR |  |  |  | B01AC01-03, B01AC05, B01AC07-56 |  |
| Acetylsalicylic acid | NPR |  |  |  | B01AC06, N02BA01 |  |
| NSAIDs | NPR |  |  |  | M01A |  |
| Combined oral contraceptives | NPR |  |  |  | G03AA, G03AB |  |
| Gestagen only contraceptives, systemic | NPR |  |  |  | G03C |  |
|  |  |  |  |  |  |  |
