# Supplementary material for: Migraine and risk of premature myocardial infarction and stroke among men and women: A Danish population-based cohort study
Source: PLoS Med. 2023 Jun 13;20(6):e1004238. doi: 10.1371/journal.pmed.1004238 (PMC10263301; doi:10.1371/journal.pmed.1004238)
Supplement: S2 Text — Legend: Purple arrows illustrate potential biasing path and green arrows illustrate causal paths. Light grey covariates indicate that we did not have information on the variable. (DOCX) [file pmed.1004238.s004.docx]

### S2 Text - Directed acyclic graphs illustrating potential confounders

#### Figure A. Directed acyclic graphs illustrating potential confounders for the analysis with myocardial infarction as the outcome.


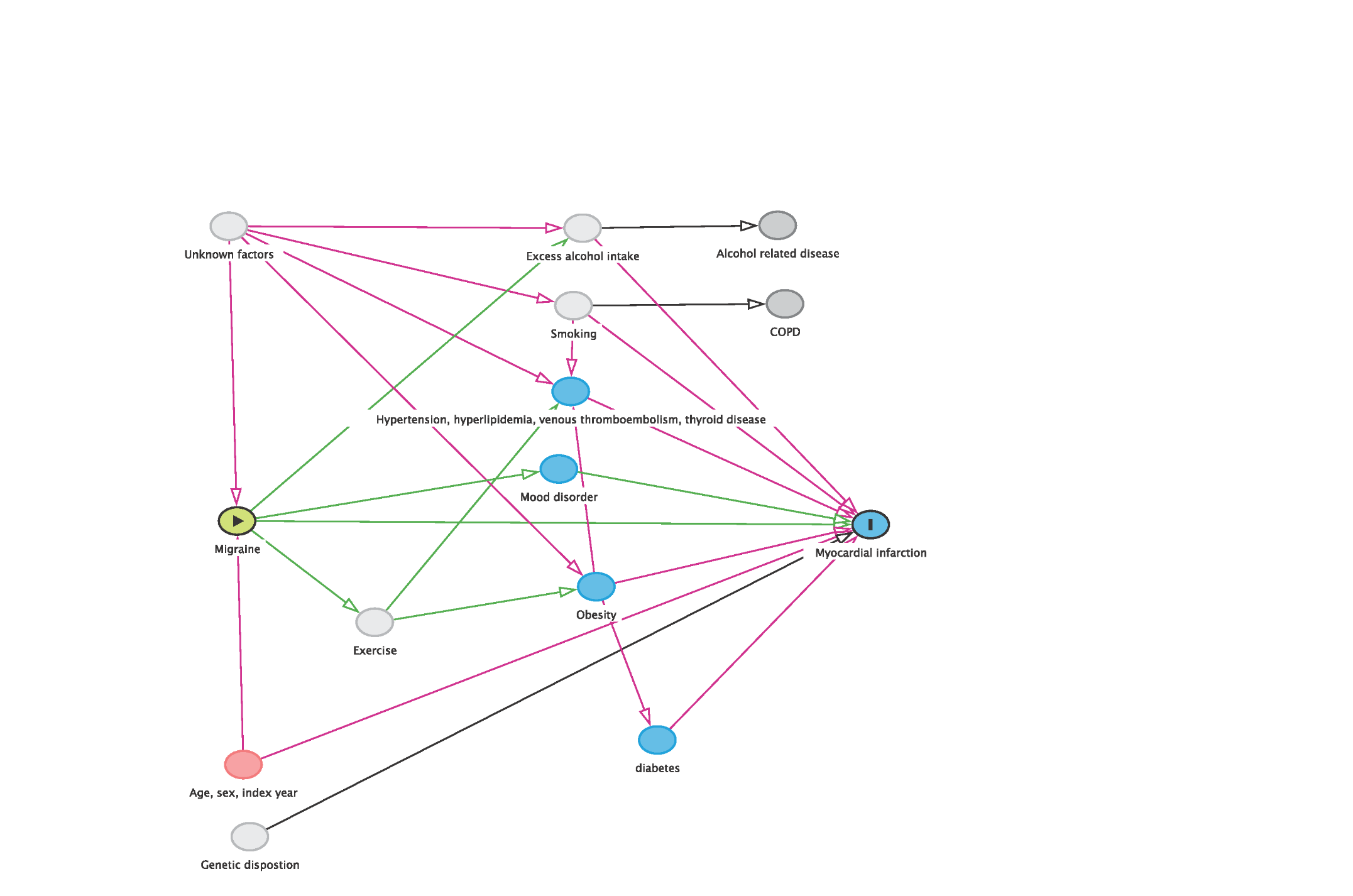


Purple arrows illustrate potential biasing path and green arrows illustrate causal paths. Light grey covariates indicate that we did not have information on the variable.

#### Figure B. Directed acyclic graphs illustrating potential confounders for the analysis with ischemic stroke as the outcome.


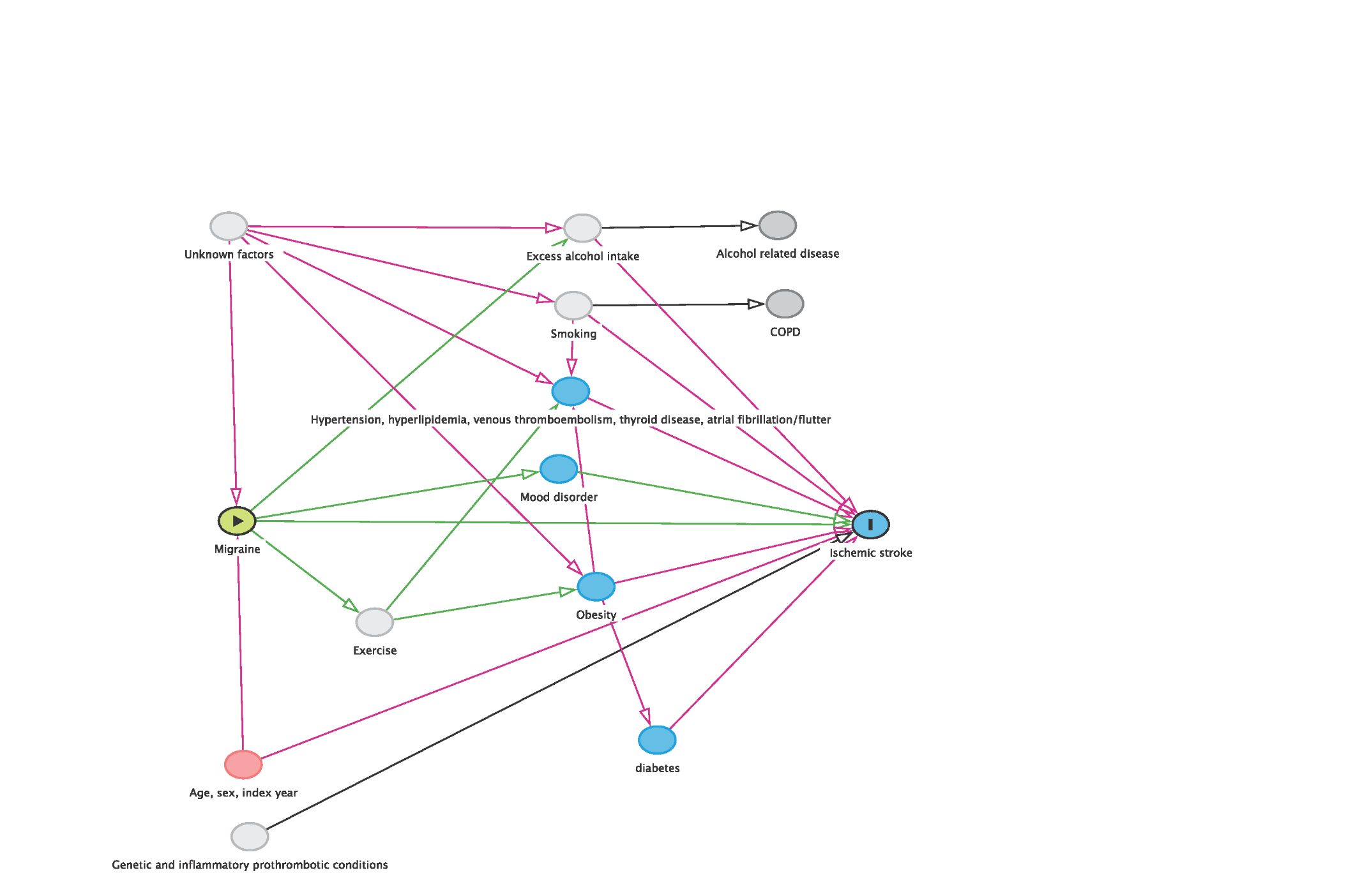


Purple arrows illustrate potential biasing path and green arrows illustrate causal paths. Light grey covariates indicate that we did not have information on the variable.

#### Figure C. Directed acyclic graphs illustrating potential confounders for the analysis with hemorrhagic stroke as the outcome.


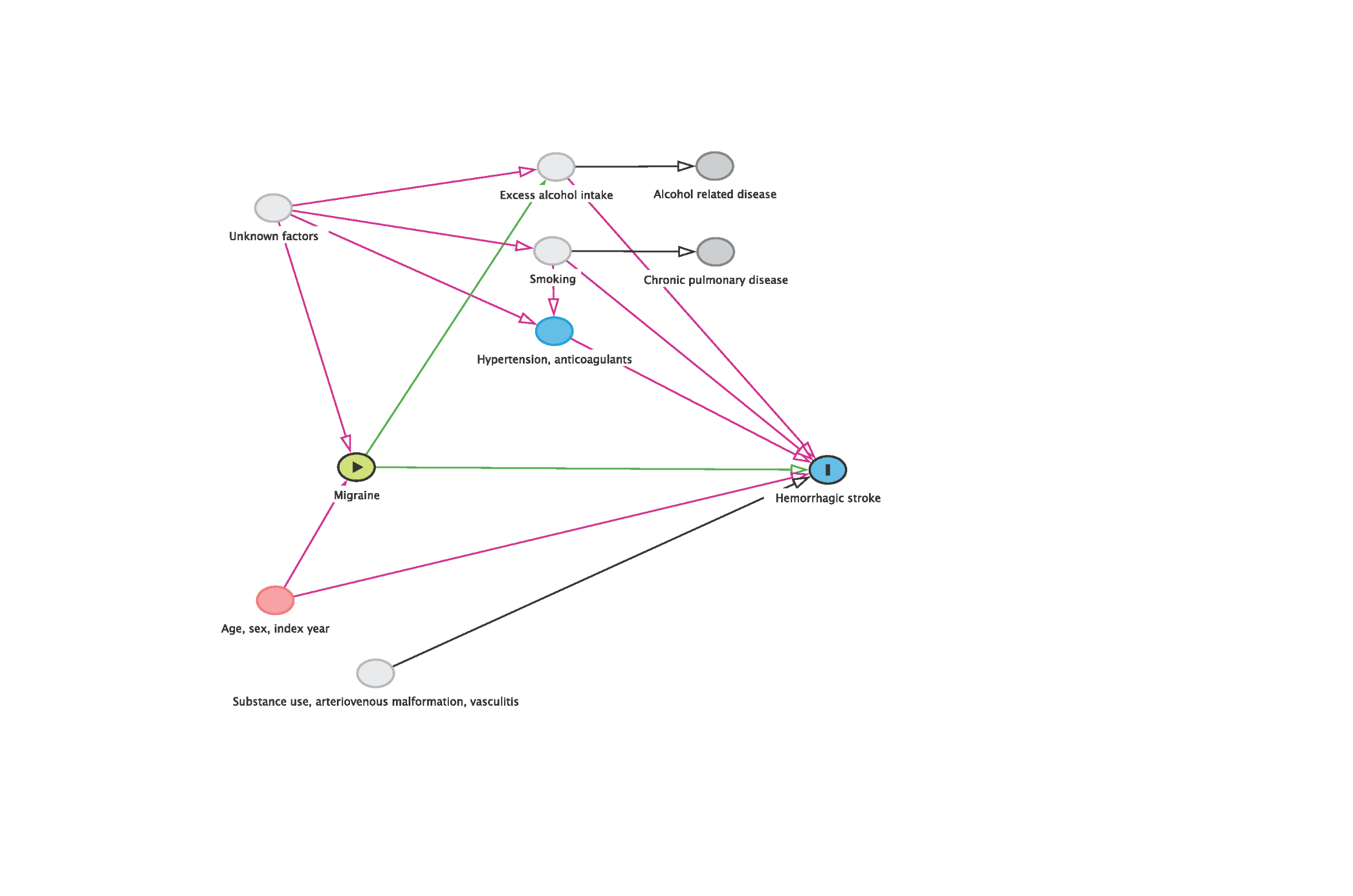


Purple arrows illustrate potential biasing path and green arrows illustrate causal paths. Light grey covariates indicate that we did not have information on the variable.
