# Supplementary material for: Migraine and risk of premature myocardial infarction and stroke among men and women: A Danish population-based cohort study
Source: PLoS Med. 2023 Jun 13;20(6):e1004238. doi: 10.1371/journal.pmed.1004238 (PMC10263301; doi:10.1371/journal.pmed.1004238)

### S3 Text.

#### Fig A. Log-log plots to assess proportional hazards assumption for cox proportional hazards model with myocardial infarction as the outcome
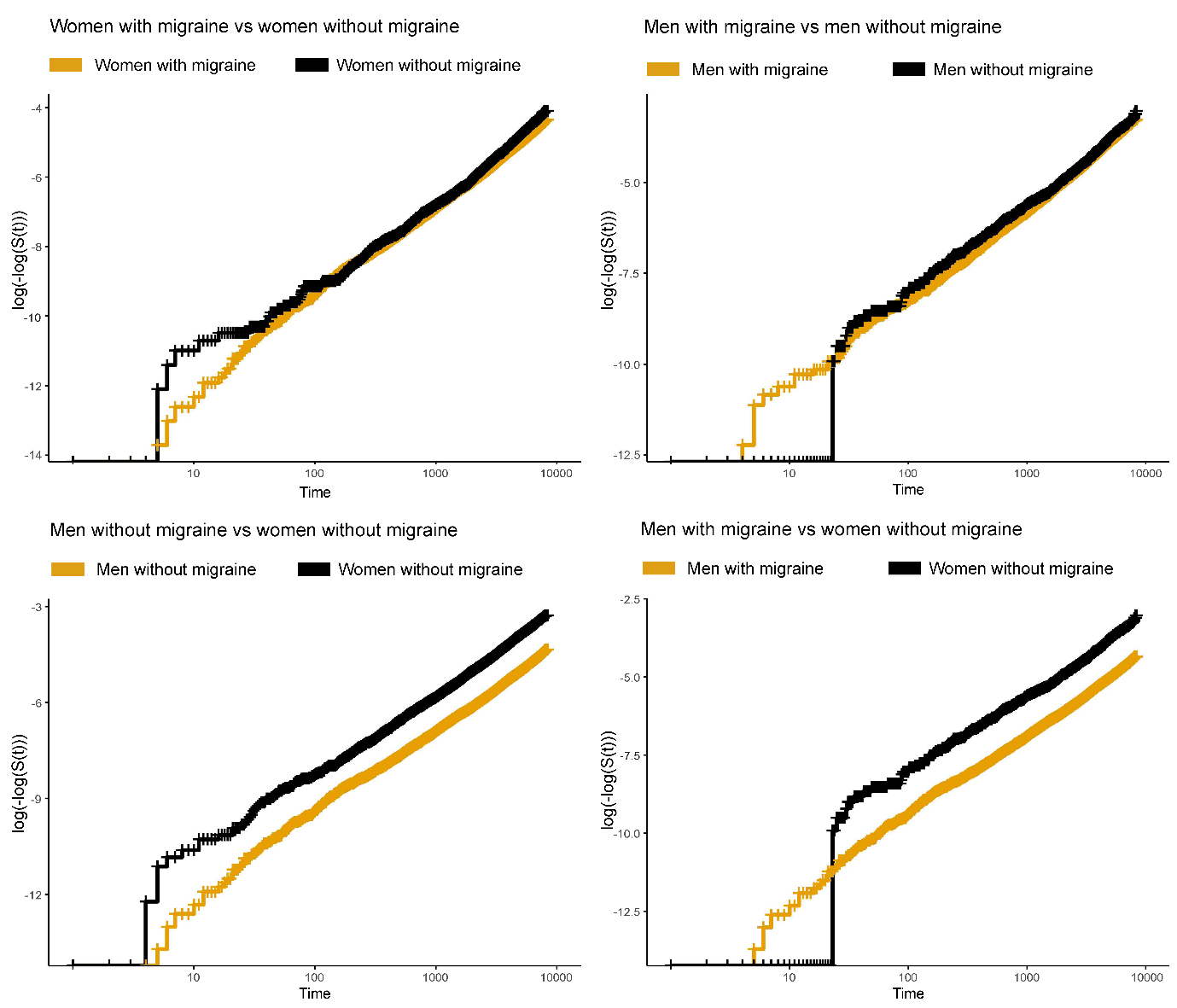


#### Fig B. Log-log plots to assess proportional hazards assumption for cox proportional hazards model with ischemic stroke as the outcome


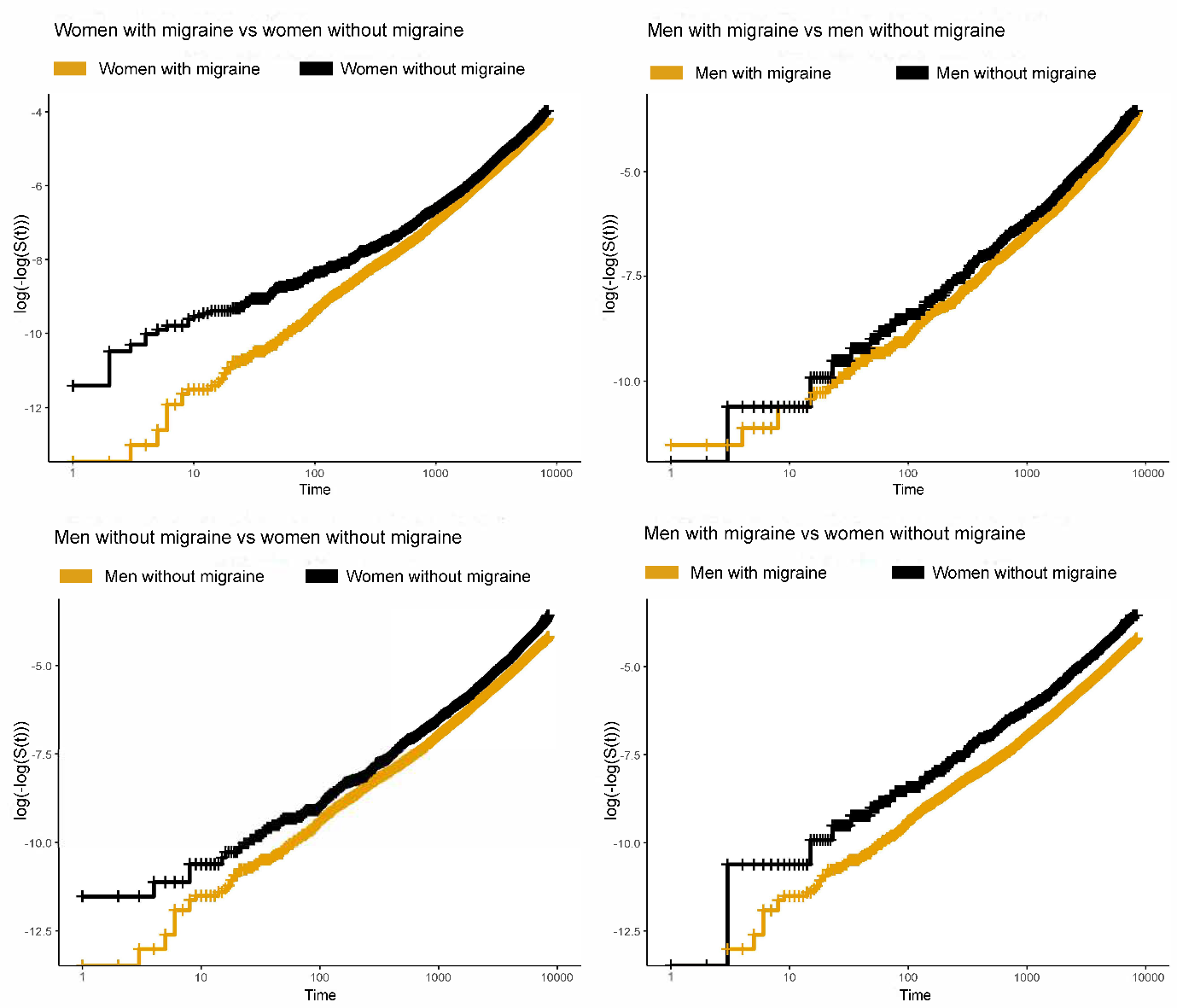


#### Fig C. Log-log plots to assess proportional hazards assumption for cox proportional hazards model with hemorrhagic stroke as the outcome.
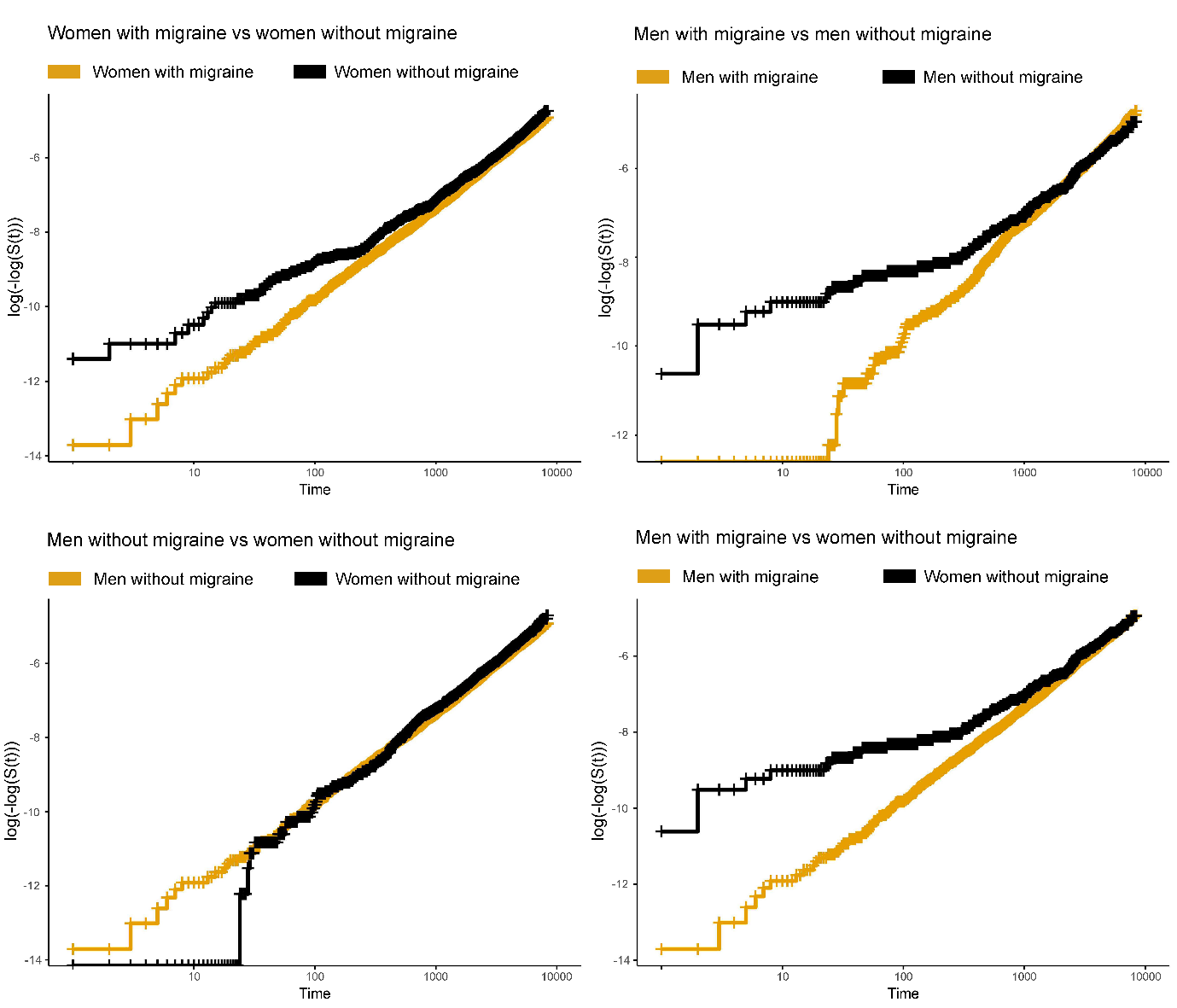


#### Fig D. Log-log plots to assess proportional hazards assumption for cox proportional hazards model with ischemic stroke including International Classification of Diseases code I64 as the outcome (sensitivity analysis 1).


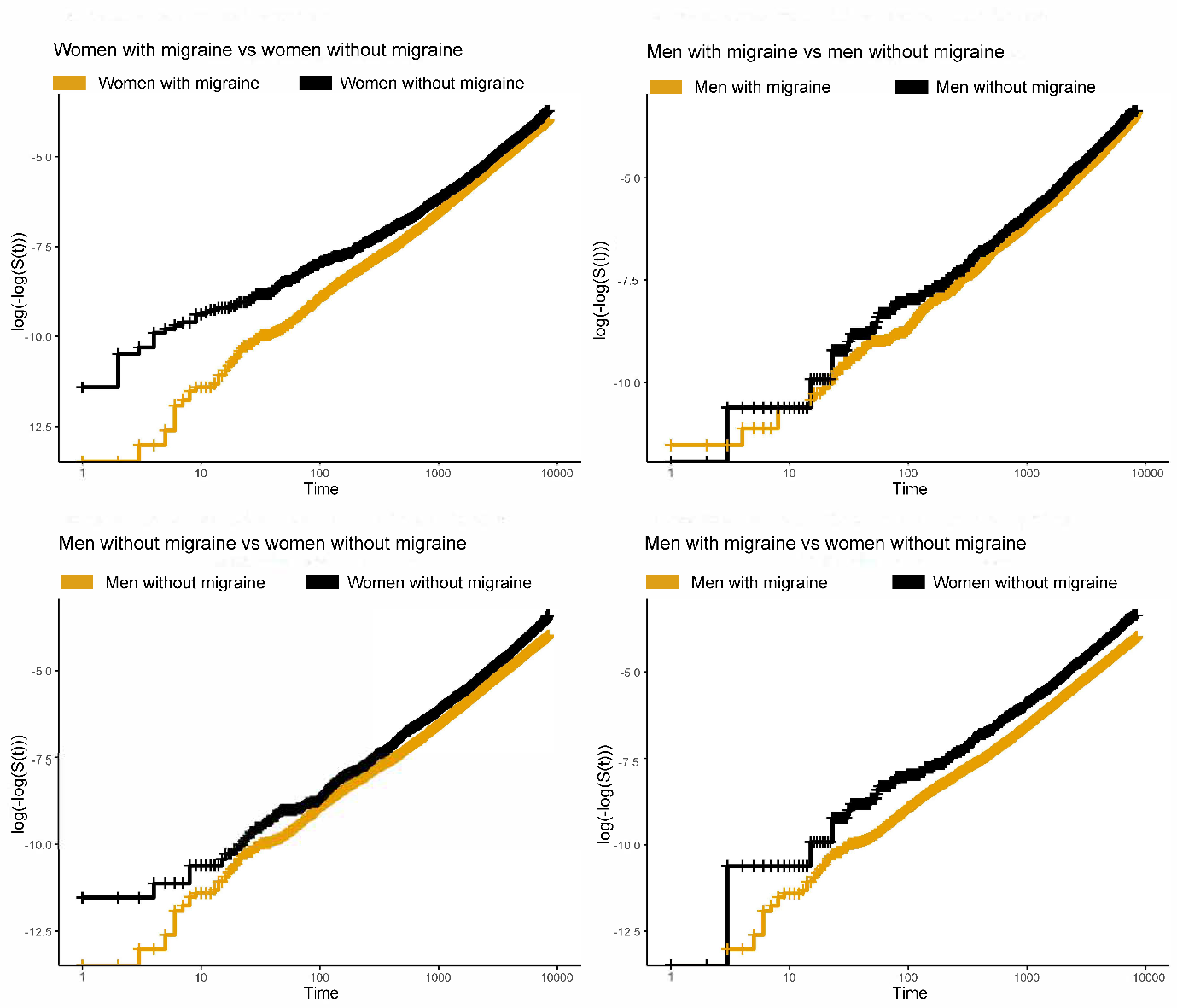


#### Fig E. Log-log plots to assess proportional hazards assumption for cox proportional hazards model with myocardial infarction as the outcome - identifying migraine by diagnostic codes in the Danish National Patient Registry (sensitivity analysis 2).


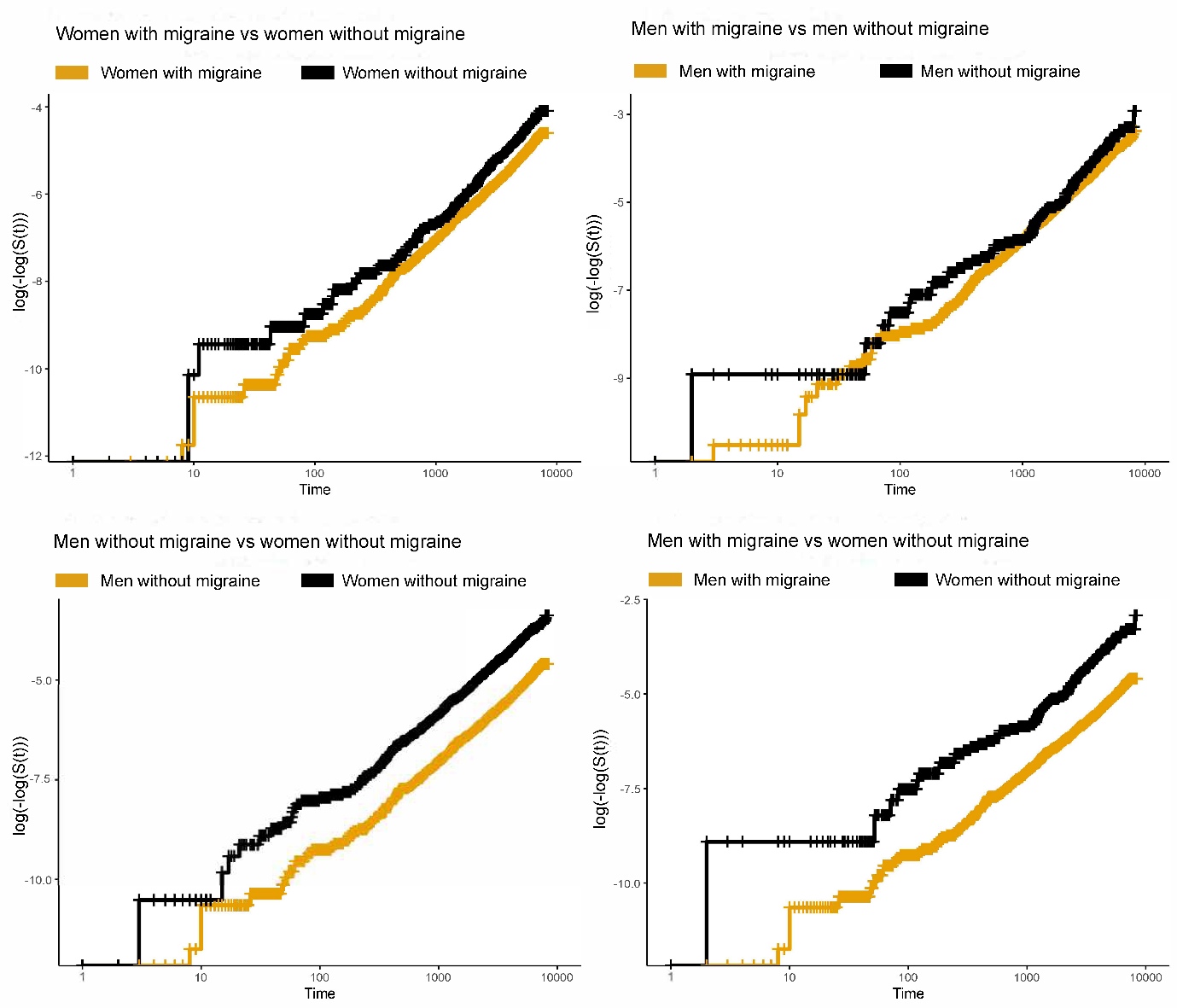


#### Fig F. Log-log plots to assess proportional hazards assumption for cox proportional hazards model with ischemic stroke as the outcome - identifying migraine by diagnostic codes in the Danish National Patient Registry (sensitivity analysis 2).


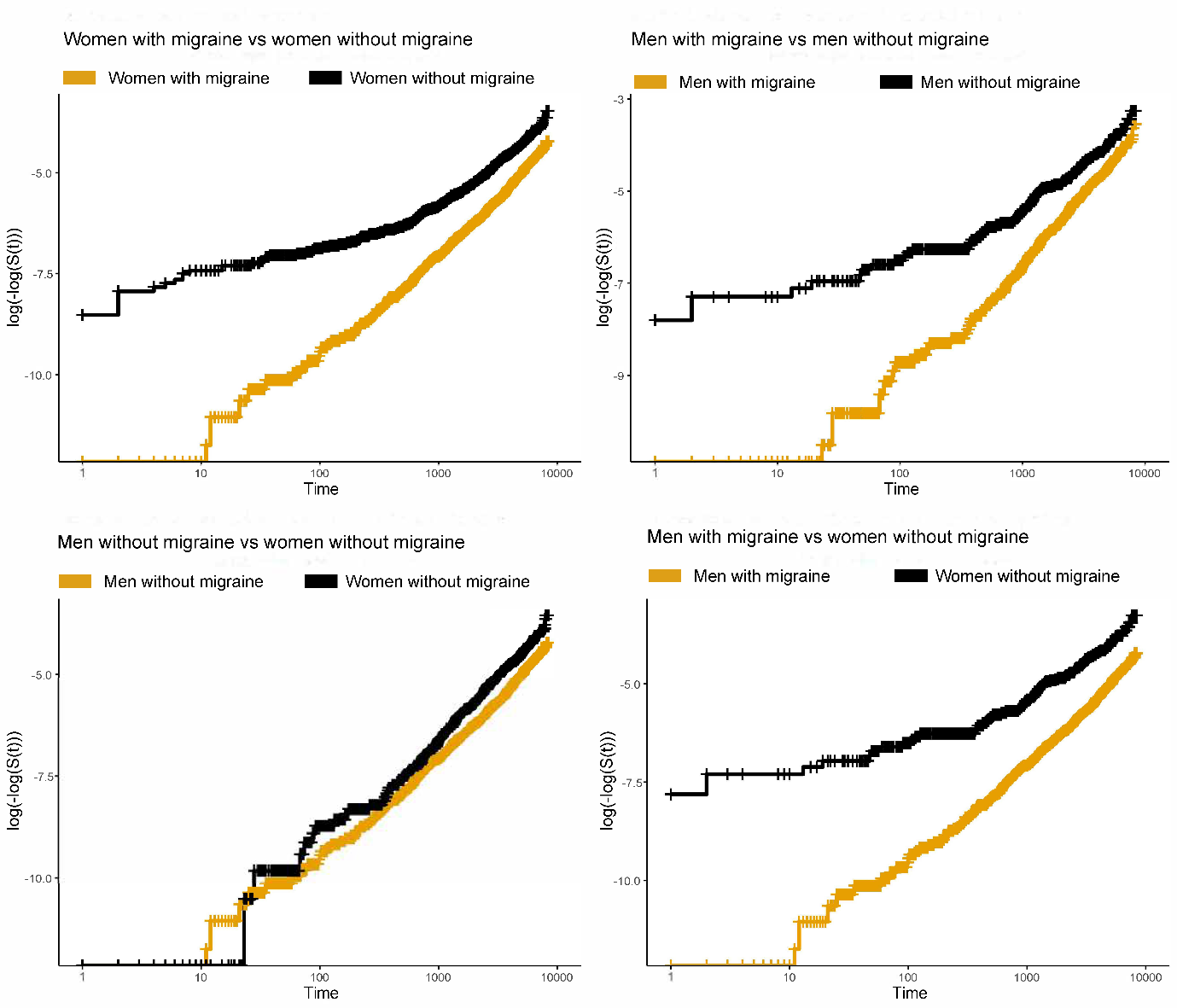


#### Fig G. Log-log plots to assess proportional hazards assumption for cox proportional hazards model with hemorrhagic stroke as the outcome - identifying migraine by diagnostic codes in the Danish National Patient Registry (sensitivity analysis 2).


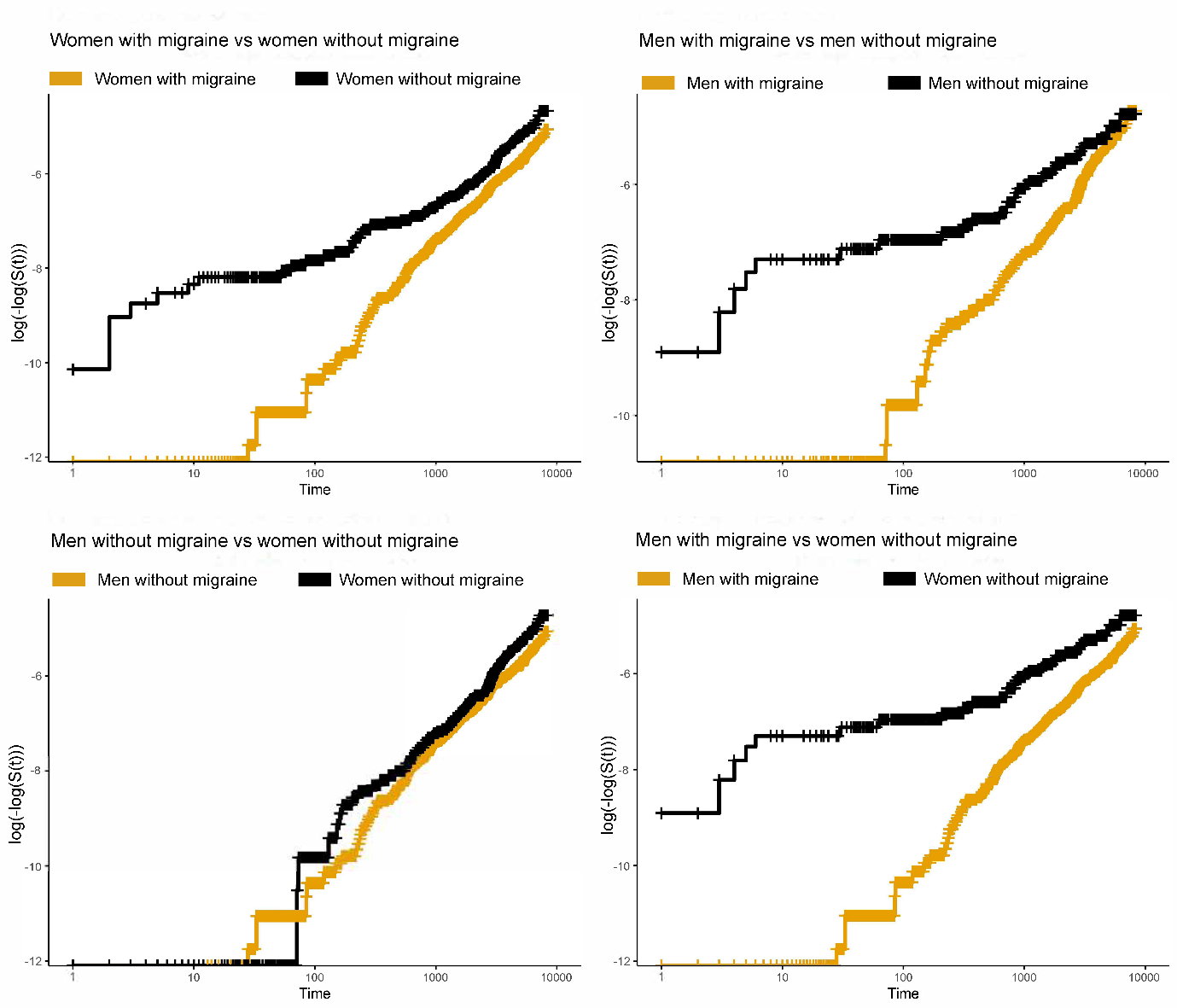

Supplement: S3 Text — (DOCX) [file pmed.1004238.s005.docx]
