# Supplementary material for: Migraine and risk of premature myocardial infarction and stroke among men and women: A Danish population-based cohort study
Source: PLoS Med. 2023 Jun 13;20(6):e1004238. doi: 10.1371/journal.pmed.1004238 (PMC10263301; doi:10.1371/journal.pmed.1004238)
Supplement: S4 Text — (DOCX) [file pmed.1004238.s006.docx]

### S4 Text. ICD codes and ATC codes for exposure, outcomes, and covariates.

| **Table A. Definition of exposures and outcomes.** | | | |
| --- | --- | --- | --- |
| **Variable** | **ICD-8^a^ codes** | **ICD-10^a^ codes** | **ATC^b^codes** |
| **Migraine in main analysis** |  |  | At least two redemptions: N02CC, N02CA01-02,  N02CA04, N02CA52  N02CX01-02 |
| **Migraine in sensitivity analysis** | 346.00  346.08  346.09 | G43.0-43.3 (excluding G43.3A which is migraine with cerebral infarction)  G43.8  G43.9 |  |
| Migraine with aura | - | G43.1 |  |
| Migraine without aura | - | G43.0 |  |
| Other migraine | - | G43.2, G43.3 (excluding G43.3A), G43.8, G43.9 |  |
| **Ischemic stroke, specified** | 433-434 | I63 |  |
| **Ischemic stroke and unspecified stroke** | 433-434 | I63-64 |  |
| **Hemorrhagic stroke** | 430-431 | I60-61 |  |
| **Myocardial infarction** | 410 | I21 |  |
| ^a^ World Health Organization’s *International Classification of Diseases*, *Eighth Revision*, (ICD-8), and *Tenth^h^ Revision* (ICD-10).  ^b^ Anatomical Therapeutic Chemical Classification System. | | | |

| **Table B. Codes used to identify comorbidities and redeemed prescriptions.** | | | | |
| --- | --- | --- | --- | --- |
| **Variable** | **ICD-8^a^ codes** | **ICD-10^a^ codes** | **ATC^b^ codes** | **Notes** |
| Atrial fibrillation or flutter | 427.93  427.94 | I48 |  |  |
| Hypertension | 400-404 | I10-15 |  |  |
| Hyperlipidemia based on diagnosis or lipid-lowering drug | 272.00, 272.01, 272.08, 272.09 | E78.0-E78.5 | C10 |  |
| Pulmonary embolism | 450.99 | I26 |  |  |
| Deep vein thrombosis | 451.00 | I80.1-I80.3 |  |  |
| Valvular heart disease | 394, 395 | I05, I06, I34, I35, I39, I39.1, I51.1A |  |  |
| Raynaud’s disease | 443.00, 443.01, 443.08, 443.09 | I73.0 |  |  |
| Obesity | 277 | E65-E66 |  |  |
| Thyroid disease | 240-246 | E00-E07 |  |  |
| Alcoholism-related disease | 291, 303, 577.10 | F10 (except F10.0), Z72.1, K86.0, G31.2, G62.1, G72.1, I42.6, K29.2, Z71.4 | Before 31 December 2000: V03AA  After 1 January 2001:  N07BB | Either ICD code or ATC code |
| Mood disorder | 296.x9 (excluding 296.89), 298.09, 298.19, 300.49, 301.19 | F30-F39 |  |  |
| Beta-blockers |  |  | C07 |  |
| ACE-inhibitor/ATII-receptor blocker^c^ |  |  | C09A, C09B, C09C, C09D |  |
| Diuretics |  |  | C03 |  |
| Vitamin K antagonists |  |  | B01AA |  |
| DOAC^d^ |  |  | B01AE, B01AF |  |
| Platelet inhibitor including clopidogrel and low-dose aspirin |  |  | B01AC04,B01AC01-03, B01AC05, B01AC07-56, B01AC06, |  |
| NSAIDs^e^ |  |  | M01A |  |
| Combined oral contraceptives |  |  | G03AA, G03AB |  |
| Systemic progesterone-only contraceptives |  |  | G03C |  |
| ^a^ World Health Organization’s *International Classification of Diseases*, *Eighth Revision*, (ICD-8), and *Tenth Revision* (ICD-10).  ^b^ Anatomical Therapeutic Chemical Classification System.  ^c^ ACE inhibitor/ AT II-antagonist: Angiotensin-Converting Enzyme inhibitor/Angiotensin II antagonist.  ^d^ Direct oral anticoagulants.  ^e^ Non-steroidal Anti-Inflammatory Drugs. | | | | |

| **Table C. Definition of comorbidities in the Charlson Comorbidity Index.** | | | |
| --- | --- | --- | --- |
|  | **ICD-8^a^ codes** | **ICD-10^a^ codes** | **Score for calculating index** |
| Heart failure | 427.09, 427.10, 427.11, 427.19, 428.99, 782.49 | I50, I11.0, I13.0, I13.2 | Score: 1 |
| Peripheral vascular disease | 440, 441, 442, 443, 444, 445 | I70, I71, I72, I73, I74, I77 | Score: 1 |
| Dementia | 290.09-290.19, 293.09 | F00-F03, F05.1, G30 | Score: 1 |
| Chronic pulmonary disease | 490-493, 515-518 | J40-J47, J60-J67, J68.4, J70.1,  J70.3, J84.1, J92.0, J96.1, J98.2, J98.3 | Score: 1 |
| Connective tissue disease | 712, 716, 734, 446, 135.99 | M05, M06, M08, M09, M30, M31,  M32, M33, M34, M35, M36, D86 | Score: 1 |
| Ulcer disease | 530.91, 530.98, 531-534 | K22.1, K25-K28 | Score: 1 |
| Mild liver disease | 571, 573.01, 573.04 | B18, K70.0-K70.3, K70.9, K71, K73, K74, K76.0 | Score: 1 |
| Diabetes | 249.00, 249.06, 249.07, 249.09, 250.00, 250.06, 250.07, 250.09 | E10.0, E10.1, E10.9,  E11.0, E11.1, E11.9 | Score: 1 |
| Hemiplegia | 344 | G81, G82 | Score: 2 |
| Moderate to severe renal disease | 403, 404, 580-583, 584, 590.09, 593.19, 753.10-753.19, 792 | I12, I13, N00-N05, N07, N11, N14, N17-N19, Q61 | Score: 2 |
| Diabetes with end- organ damage | 249.01-249.05, 249.08,  250.01-250.05, 250.08 | E10.2-E10.8  E11.2-E11.8 | Score: 2 |
| Any tumor | 140-194 | C00-C75 | Score: 2 |
| Leukemia | 204-207 | C91-C95 | Score: 2 |
| Lymphoma | 200-203, 275.59 | C81-C85, C88, C90, C96 | Score: 2 |
| Moderate to severe liver disease | 070.00, 070.02, 070.04, 070.06, 070.08, 573.00, 456.00-456.09 | B15.0, B16.0, B16.2, B19.0, K70.4, K72, K76.6, I85 | Score: 3 |
| Metastatic solid tumor | 195-198, 199 | C76-C80 | Score: 6 |
| AIDS | 079.83 | B21-B24 | Score: 6 |
| ^a^World Health Organization’s *International Classification of Diseases*, *Eighth Revision*, (ICD-8), and *Tenth Revision* (ICD-10). | | | |
