# Supplementary material for: Migraine and risk of premature myocardial infarction and stroke among men and women: A Danish population-based cohort study
Source: PLoS Med. 2023 Jun 13;20(6):e1004238. doi: 10.1371/journal.pmed.1004238 (PMC10263301; doi:10.1371/journal.pmed.1004238)
Supplement: S5 Text — (DOCX) [file pmed.1004238.s007.docx]

### S5 Text. Sensitivity analysis 2: Identifying migraine using diagnosis codes in the DNPR.

| Table A. Baseline characteristics (n, %) of women and men with and without migraine, identifying migraine using diagnosis codes. | | | | | | | |
| --- | --- | --- | --- | --- | --- | --- | --- |
|  | **Women** | | |  | **Men** | | |
|  | **Without migraine** |  | **With migraine** |  | **Without migraine** |  | **With migraine** |
| All | 126,365 (100) |  | 25,274 (100) |  | 36,985 (100) |  | 7397 (100) |
| Age (median, IQR) | 37.8 (29.3, 46.5) |  | 37.8 (29.3, 46.5) |  | 39.0 (30.1, 47.6) |  | 38.9 (30.1, 47.6) |
| Type of migraine | | | | | | | |
| Migraine with aura | - |  | 10,154 (40.2) |  | - |  | 3525 (47.7) |
| Migraine without aura | - |  | 13,674 (54.1) |  | - |  | 3322 (44.9) |
| Other migraine | - |  | 1446 (5.7) |  | - |  | 550 (7.4) |
| Calendar period |  |  |  |  |  |  |  |
| 1996-2000 | 14,760 (11.7) |  | 2,952 (11.7) |  | 4,800 (13.0) |  | 960 (13.0) |
| 2001-2005 | 24,675 (19.5) |  | 4,935 (19.5) |  | 6,690 (18.1) |  | 1,338 (18.1) |
| 2006-2010 | 30,995 (24.5) |  | 6,199 (24.5) |  | 9,250 (25.0) |  | 1,850 (25.0) |
| 2011-2014 | 29,750 (23.5) |  | 5950 (23.5) |  | 8305 (22.5) |  | 1661 (22.5) |
| 2015-2018 | 26,185 (20.7) |  | 5238 (20.7) |  | 7940 (21.5) |  | 1588 (21.5) |
| Highest level of education^a^ | |  |  |  |  |  |  |
| Low | 31,495 (24.9) |  | 6924 (27.4) |  | 10,030 (27.1) |  | 2129 (28.8) |
| Medium | 56,567 (44.8) |  | 11,191 (44.3) |  | 18,445, (49.9) |  | 3657 (49.4) |
| High | 32,5777 (25.8) |  | 6,336 (25.1) |  | 6,718 (18.2) |  | 1364 (18.4) |
| Unknown | 5726 (4.5) |  | 823 (3.3) |  | 1,792 (4.8) |  | 247 (3.3) |
| Level of income^b^ |  |  |  |  |  |  |  |
| Low | 37,644 (29.8) |  | 7,689 (30.4) |  | 10,434 (28.2) |  | 2099 (28.4) |
| Medium | 35,695 (28.2) |  | 7708 (30.5) |  | 10,551 (28.5) |  | 2150 (29.1) |
| High | 29,790 (23.6) |  | 5616 (22.2) |  | 8,422 (22.8) |  | 1683 (22.8) |
| Very high | 22,349 (17.7) |  | 4199 (16.6) |  | 7332 (19.8) |  | 1454 (19.7) |
| Missing | 887 (0.7) |  | 62 (0.2) |  | 246 (0.7) |  | 11 (0.1) |
| Charlson Comorbidity Index score^c^ | |  |  |  |  |  |  |
| Low | 113,330 (89.7) |  | 21,386 (84.6) |  | 33,360 (90.2) |  | 6323 (85.5) |
| Medium | 11,733 (9.3) |  | 3517 (13.9) |  | 3207 (8.7) |  | 947 (12.8) |
| High | 1302 (1.0) |  | 371 (1.5) |  | 418(1.1) |  | 127 (1.7) |
| ^a^ Low= primary or lower secondary education, medium= upper secondary or academic profession degree, high= university education at bachelor degree or higher.  ^b^ According to quartiles. Quartiles were calculated separately for women and men.  ^c^ Charlson Comorbidity Index Score: low= 0 (no comorbidities), medium= 1-2, and high= 3 or more. | | | | | | | |

####
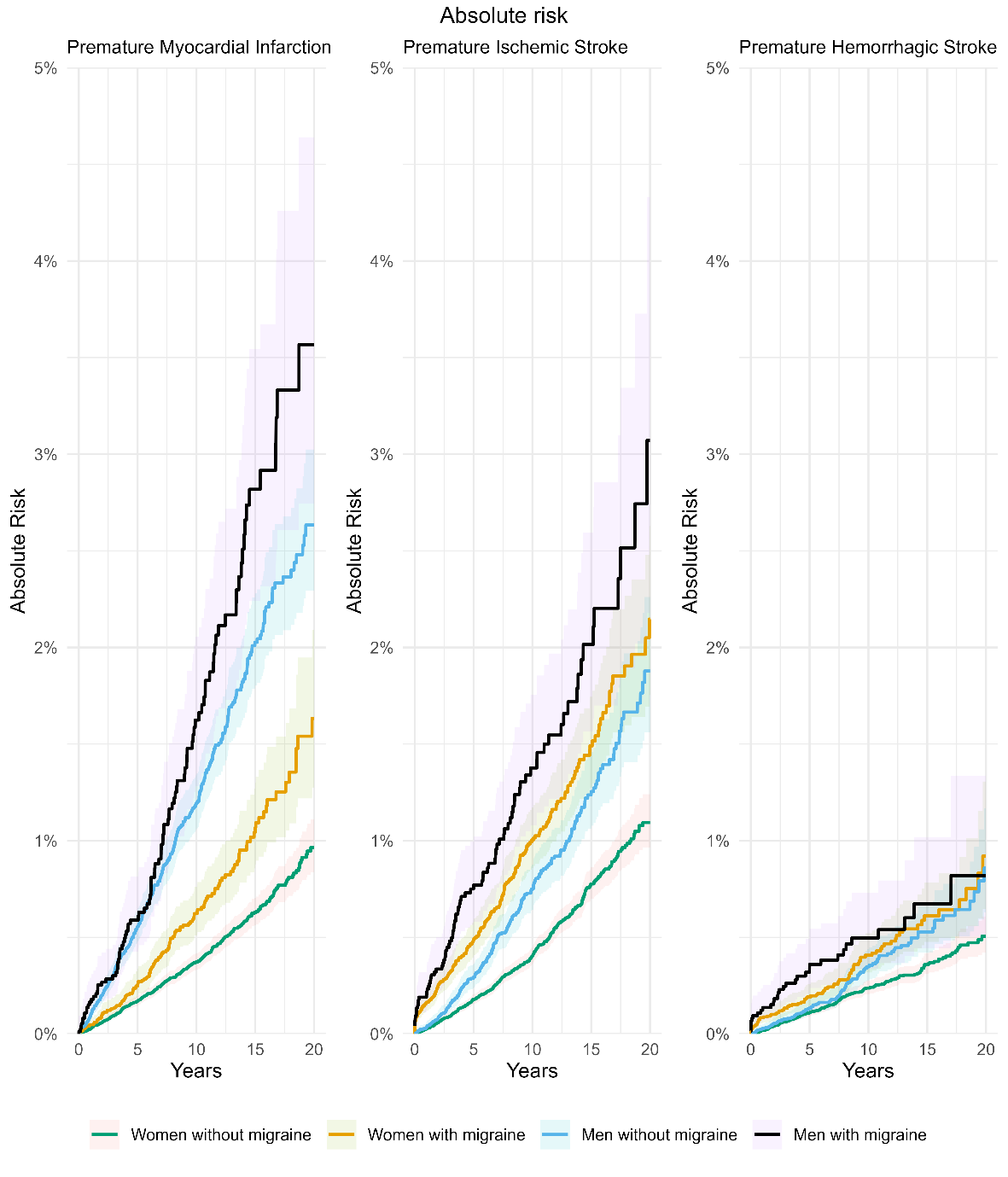


**S5 Text Fig A. Absolute risk of premature myocardial infarction, ischemic stroke, and hemorrhagic stroke when migraine was identified by diagnosis codes.**

The 95% confidence intervals illustrated by the shadowed areas. Difference in impact of migraine was evaluated for men and women separately using Gray’s test. For myocardial infarction, the p-value was <0.001 for women and 0.018 for men. For ischemic stroke, the p-value was <0.001 for women and <0.001 for men. For hemorrhagic stroke the p-value was <0.001 for women and 0.035 for men.

| Table B. Absolute risks, risk differences (RDs), crude and adjusted hazard ratios (HRs) for 1–20 years of follow-up for premature myocardial infarction, ischemic stroke, and hemorrhagic stroke for women and men with and without migraine (identified by diagnostic codes). P-values reflect Gray’s test for RDs and likelihood ratio tests for HRs. | | | | | | | | |
| --- | --- | --- | --- | --- | --- | --- | --- | --- |
|  | **Events**  **n** | **Absolute risk**  **% (95% CI)** | **RD within sex**  **% (95% CI)** | **Crude HR**  **(95% CI)** | **Crude HR within sex**  **(95% CI)** | **Adjusted* HR**  **(95% CI)** | **Adjusted* HR within sex**  **(95% CI)** |  |
| Myocardial infarction |  |  |  |  |  |  |  |  |
| Women without migraine | 398 | 0.9 (0.8, 1.1) | - | 1.00 (ref) | 1.00 (ref) | 1.00 (ref) | 1.00 (ref) |  |
| Women with migraine | 131 | 1.6 (1.2, 2.0) | 0.7 (0.2,1.1); p<0.001 | 1.63 (1.34, 1.99); p<0.001 | 1.63 (1.34, 1.99); p<0.001 | 1.58 (1.30, 1.93); p<0.001 | 1.58 (1.30, 1.93); p<0.001 |  |
| Men without migraine | 354 | 2.5 (2.2, 2.9) | - | 3.15 (2.73; 3.63); p<0.001 | 1.00 (ref) | 2.99 (2.59, 3.46); p<0.001 | 1.00 (ref) |  |
| Men with migraine | 89 | 3.4 (2.5, 4.4) | 0.8 (0.2, 1.8); p=0.048 | 3.97 (3.15, 4.99); p<0.001 | 1.26 (1.00, 1.59); p=0.051 | 3.52 (2.79, 4.44); p<0.001 | 1.20 (0.95, 1.51); p=0.131 |  |
| Ischemic stroke |  |  |  |  |  |  |  |  |
| Women without migraine | 455 | 1.1 (0.9, 1.2) | - | 1.00 (ref) | 1.00 (ref) | 1.00 (ref) | 1.00 (ref) |  |
| Women with migraine | 180 | 2.0 (1.6, 2.4) | 0.9 (0.5, 1.4); p<0.001 | 1.97 (1.66, 2.34); p<0.001 | 1.97 (1.66, 2.34); p<0.001 | 1.91 (1.61, 2.27); p<0.001 | 1.91 (1.61, 2.27); p<0.001 |  |
| Men without migraine | 226 | 1.8 (1.5, 2.2) | - | 1.75 (1.49, 2.05); p<0.001 | 1.00 (ref) | 1.64 (1.40, 1.93); p<0.001 | 1.00 (ref) |  |
| Men with migraine | 73 | 2.9 (1.8, 3.9) | 1.0 (-0.1, 2.1); p<0.001 | 2.84 (2.22, 3.64); p<0.001 | 1.62 (1.25, 2.11); p<0.001 | 2.54 (1.98, 3.25); p<0.001 | 1.55 (1.19, 2.02); p=0.001 |  |
| Hemorrhagic stroke |  |  |  |  |  |  |  |  |
| Women without migraine | 231 | 0.5 (0.4, 0.6) | - | 1.00 (ref) | 1.00 (ref) | 1.00 (ref) | 1.00 (ref) |  |
| Women with migraine | 67 | 0.8 (0.5, 1.2) | 0.3 (0.0, 0.7); p=0.011 | 1.44 (1.10, 1.89); p=0.009 | 1.44 (1.10, 1.89); p=0.009 | 1.40 (1.07, 1.84); p=0.015 | 1.40 (1.07, 1.84); p=0.015 |  |
| Men without migraine | 95 | 0.8 (0.6, 1.1) | - | 1.45 (1.14,1.84); p=0.002 | 1.00 (ref) | 1.32 (1.03, 1.68); p=0.026 | 1.00 (ref) |  |
| Men with migraine | 23 | 0.7 (0.3, 1.1) | -0.1 (-0.6, 0.4); p=0.403 | 1.75 (1.14, 2.69); p=0.10 | 1.21 (0.77, 1.91); p=0.404 | 1.55 (1.00, 2.39); p=0.046 | 1.16 (0.73, 1.83); p=0.534 |  |
| * For MI: adjusted for age, calendar period, hypertension, thyroid disease, hyperlipidemia, VTE, obesity, alcohol-related disease, and COPD.  For ischemic stroke: adjusted for age, calendar period, hypertension, thyroid disease, hyperlipidemia, VTE, obesity, alcohol-related disease, COPD and atrial fibrillation/flutter.  For hemorrhagic stroke: adjusted for age, calendar period, hypertension, alcohol-related disease, COPD, and anticoagulant treatment. | | | | | | | |  |
