# Supplementary material for: Migraine and risk of premature myocardial infarction and stroke among men and women: A Danish population-based cohort study
Source: PLoS Med. 2023 Jun 13;20(6):e1004238. doi: 10.1371/journal.pmed.1004238 (PMC10263301; doi:10.1371/journal.pmed.1004238)
Supplement: S1 Table — (DOCX) [file pmed.1004238.s008.docx]

### S1 Table. Sensitivity analysis 1: Risk of ischemic stroke including ICD code I64.

| Absolute risks, risk differences (RDs), crude and adjusted hazard ratios (HRs) for 1–20 years of follow-up for premature ischemic stroke (including code for unspecified stroke) for women and men with and without migraine (identified by prescription data). P-values reflect Gray’s test for RDs and likelihood ratio tests for HRs. | | | | | | | | |
| --- | --- | --- | --- | --- | --- | --- | --- | --- |
|  | **Events**  **n** | **Absolute risk**  **% (95% CI)** | **RD within sex**  **% (95% CI)** | **Crude HR**  **(95% CI)** | **Crude HR within sex**  **(95% CI)** | **Adjusted* HR**  **(95% CI)** | **Adjusted* HR within sex**  **(95% CI)** |  |
| Ischemic and unspecified stroke | | | | | | | |  |
| Women without migraine | 5495 | 1.5 (1.4, 1.5) | - | 1.00 (Ref) | 1.00 (Ref) | 1.00 (Ref) | 1.00 (Ref) |  |
| Women with migraine | 1381 | 1.9 (1.7, 2.0) | 0.4 (0.3, 0.6);  p<0.001 | 1.25 (1.18, 1.33); p<0.001 | 1.25 (1.18, 1.33); p<0.001 | 1.24 (1.17, 1.31); p<0.001 | 1.24 (1.17, 1.31); p<0.001 |  |
| Men without migraine | 1897 | 2.4 (2.2, 2.5) | - | 1.57 (1.49, 1.65); p<0.001 | 1.00 (Ref) | 1.58 (1.50, 1.66); p<0.001 | 1.00 (Ref) |  |
| Men with migraine | 465 | 2.9 (2.5, 3.2) | 0.5 (0.2, 0.9);  p<0.001 | 1.92 (1.75, 2.11); p<0.001 | 1.23 (1.11, 1.36); p<0.001 | 1.87 (1.70, 2.06); p<0.001 | 1.20 (1.08, 1.33); p<0.001 |  |
| * For MI: adjusted for age, calendar period, hypertension, thyroid disease, hyperlipidemia, VTE, obesity, alcohol-related disease, and COPD.  For ischemic stroke: adjusted for age, calendar period, hypertension, thyroid disease, hyperlipidemia, VTE, obesity, alcohol-related disease, COPD and atrial fibrillation/flutter.  For hemorrhagic stroke: adjusted for age, calendar period, hypertension, alcohol-related disease, COPD, and anticoagulant treatment. | | | | | | | |  |
