# Supplementary material for: Migraine and risk of premature myocardial infarction and stroke among men and women: A Danish population-based cohort study
Source: PLoS Med. 2023 Jun 13;20(6):e1004238. doi: 10.1371/journal.pmed.1004238 (PMC10263301; doi:10.1371/journal.pmed.1004238)
Supplement: S2 Table — (DOCX) [file pmed.1004238.s009.docx]

### S2 Table. Sensitivity analysis 3: 1-10 year hazard ratios stratified by index year (1996-2006, 2007-2018)

| Absolute risks, risk differences (RDs), crude and adjusted hazard ratios (HRs) for 1–10 years of follow-up for premature myocardial infarction, ischemic stroke, and hemorrhagic stroke stratified by index year (migraine identified by prescriptions). P-values reflect likelihood ratio test. | | | | |
| --- | --- | --- | --- | --- |
|  | **Crude HR**  **(95% CI)** | **Crude HR within sex**  **(95% CI)** | **Adjusted* HR**  **(95% CI)** | **Adjusted* HR within sex**  **(95% CI)** |
| Myocardial infarction | | | | |
| 1996–2006 |  |  |  |  |
| Women without migraine | 1.00 (Ref) | 1.00 (Ref) | 1.00 (Ref) | 1.00 (Ref) |
| Women with migraine | 1.15 (1.03, 1.29); p=0.013 | 1.15 (1.03, 1.29); p=0.013 | 1.14 (1.02, 1.27); p=0.019 | 1.14 (1.02, 1.27); p=0.019 |
| Men without migraine | 3.13 (2.90, 3.38); p<0.001 | 1.00 (Ref) | 3.31 (3.07, 3.58); p<0.001 | 1.00 (Ref) |
| Men with migraine | 3.07 (2.67, 3.54); p<0.001 | 0.98 (0.85, 1.13); p=0.800 | 3.15 (2.73, 3.63); p<0.001 | 0.97 (0.84, 1.12); p=0.649 |
| 2007–2018 |  |  |  |  |
| Women without migraine | 1.00 (Ref) | 1.00 (Ref) | 1.00 (Ref) | 1.00 (Ref) |
| Women with migraine | 1.18 (1.02, 1.39); p=0.029 | 1.18 (1.02, 1.39); p=0.029 | 1.16 (0.99, 1.35); p=0.068 | 1.16 (0.99, 1.35); p=0.068 |
| Men without migraine | 2.91 (2.62, 3.24); p<0.001 | 1.00 (Ref) | 3.11 (2.79, 3.47); p<0.001 | 1.00 (Ref) |
| Men with migraine | 3.76 (3.14, 4.49); p<0.001 | 1.29 (1.07, 1.55); p=0.007 | 3.87 (3.23, 4.63); p<0.001 | 1.25 (1.04, 1.51); p=0.016 |
| Ischemic stroke | | | | |
| 1996–2006 |  |  |  |  |
| Women without migraine | 1.00 (Ref) | 1.00 (Ref) | 1.00 (Ref) | 1.00 (Ref) |
| Women with migraine | 1.23 (1.11, 1.37); p<0.001 | 1.23 (1.11, 1.37); p<0.001 | 1.22 (1.10, 1.37); p<0.001 | 1.22 (1.10, 1.37); p<0.001 |
| Men without migraine | 1.57 (1.43, 1.73); p<0.001 | 1.00 (Ref) | 1.62 (1.47, 1.78); p<0.001 | 1.00 (Ref) |
| Men with migraine | 2.01 (1.69, 2.39); p<0.001 | 1.28 (1.06, 1.54); p=0.009 | 2.02 (1.70, 2.40); p<0.001 | 1.25 (1.03, 1.51); p=0.018 |
| 2007–2018 |  |  |  |  |
| Women without migraine | 1.00 (Ref) | 1.00 (Ref) | 1.00 (Ref) | 1.00 (Ref) |
| Women with migraine | 1.26 (1.10, 1.45); p<0.001 | 1.26 (1.10, 1.45); p<0.001 | 1.23 (1.07, 1.41); p=0.003 | 1.23 (1.07, 1.41); p=0.003 |
| Men without migraine | 1.41 (1.24, 1.59); p<0.001 | 1.00 (Ref) | 1.45 (1.28, 1.65); p<0.001 | 1.00 (Ref) |
| Men with migraine | 1.86 (1.49, 2.31); p<0.001 | 1.32 (1.04, 1.67); p=0.021 | 1.83 (1.47, 2.27); p<0.001 | 1.29 (1.02, 1.63); p=0.035 |
| Hemorrhagic stroke | | | | |
| 1996–2006 |  |  |  |  |
| Women without migraine | 1.00 (Ref) | 1.00 (Ref) | 1.00 (Ref) | 1.00 (Ref) |
| Women with migraine | 1.11 (0.96, 1.27); p=0.164 | 1.11 (0.96, 1.27); p=0.164 | 1.10 (0.96, 1.27); p=0.180 | 1.10 (0.96, 1.27); p=0.180 |
| Men without migraine | 1.15 (1.01, 1.32); p=0.041 | 1.00 (Ref) | 1.14 (1.00, 1.31); p=0.058 | 1.00 (Ref) |
| Men with migraine | 0.99 (0.73, 1.33); p=0.947 | 0.86 (0.63, 1.18) ; p=0.347 | 0.96 (0.71, 1.29); p=0.786 | 0.84 (0.61, 1.15); p=0.281 |
| 2007–2018 |  |  |  |  |
| Women without migraine | 1.00 (Ref) | 1.00 (Ref) | 1.00 (Ref) | 1.00 (Ref) |
| Women with migraine | 1.03 (0.84, 1.27); p=0.773 | 1.03 (0.84, 1.27); p=0.773 | 1.02 (0.83, 1.26); p=0.843 | 1.02 (0.83, 1.26); p=0.843 |
| Men without migraine | 1.11 (0.92, 1.35); p=0.280 | 1.00 (Ref) | 1.10 (0.91, 1.34); p=0.329 | 1.00 (Ref) |
| Men with migraine | 1.18 (0.81, 1.73); p=0.389 | 1.06 (0.71, 1.60); p=0.768 | 1.14 (0.78, 1.67); p=0.500 | 1.04 (0.69, 1.56); p=0.853 |
| *For MI: adjusted for age, calendar period, hypertension, thyroid disease, hyperlipidemia, VTE, obesity, alcohol-related disease, and COPD.  For ischemic stroke: adjusted for age, calendar period, hypertension, thyroid disease, hyperlipidemia, VTE, obesity, alcohol-related disease, COPD and atrial fibrillation/flutter.  For hemorrhagic stroke: adjusted for age, calendar period, hypertension, alcohol-related disease, COPD, and anticoagulant treatment. | | | | |
