# Supplementary material for: Migraine and risk of premature myocardial infarction and stroke among men and women: A Danish population-based cohort study
Source: PLoS Med. 2023 Jun 13;20(6):e1004238. doi: 10.1371/journal.pmed.1004238 (PMC10263301; doi:10.1371/journal.pmed.1004238)
Supplement: Attachment — Submitted filename: Point_by_point_response_migraine.docx [file pmed.1004238.s010.docx]

## Requests from the Editors

### GENERAL

1. Please respond to all editor and reviewer requests as detailed below, in full. Please include line numbers in your revised version starting at 1 and continuing in sequence thereafter. Please ensure that the study is reported according to the STROBE guideline, and include the completed STROBE checklist as Supporting Information. Please add the following statement, or similar, to the Methods: "This study is reported as per the Strengthening the Reporting of Observational Studies in Epidemiology (STROBE) guideline (S1 Checklist)."When completing the checklist, please use section and paragraph numbers, rather than page and/or line numbers, as these often change in the event of publication.

**Reply:** We thank the Editors and Reviewers for their comments and believe we have addressed their concerns. Below, page numbers refer to those in the marked version of the manuscript.

We have added line numbers and included a STROBE checklist in the submission. We also have included the statement on reporting the paper according to the STROBE guideline (as the first sentence of the Methods section) (p5).

### ABSTRACT

2. Please structure your abstract using the PLOS Medicine headings (Background, Methods and Findings, Conclusions). Please combine the Methods and Findings sections into one section, “Methods and findings”.

**Reply:** We have now structured the Abstract according to your guidelines.

3. Abstract Background:

Please ensure that the final sentence clearly states the study question.

**Reply:** The final sentence of the Background section of the Abstract now states the following: “The aim of this study was to examine impact of migraine on the risk of premature (age ≤60 years) MI and ischemic/hemorrhagic stroke among men and women.”

4. Abstract Methods and Findings:

Please ensure that all numbers presented in the abstract are present and identical to numbers presented in the main manuscript text.

**Reply:** We have checked all the numbers in the Abstract against those presented in the manuscript text and tables to ensure that they are identical.

5. Please expand the details of your study population – number of participants, age range (and mean) how did you select them? And so on.

**Reply:** We now provide this information in several sentences added to the abstract: “Redeemed prescriptions for migraine-specific medication were used to identify women with migraine (n=179,680) and men with migraine (n=40,757). Five times as many individuals who did not use migraine-specific medications were sampled from the general population and matched to the individuals with migraine on sex, index year, and birth year. All individuals were required to be between 18 and 60 years old. Median age was 41.5 years for women and 40.3 years for men.”

6. Please detail the main outcome measures.

Please quantify the main results p values as well as 95% CIs. Please ensure that you report p as <0.001 or where higher as p=0.002, for example.

Suggest reporting statistical information as follows: “…0.3% (95% CI [-0.1%, 0.6%]; p<0.001 or p=0.002), for clarity and accessibility. Please check and amend throughout for consistency in data reporting.

**Reply:** We now have specified the main outcome measures and added the p-values as suggested. Of note, the STROBE guidelines do not recommend inclusion of such values.

7. Please detail factors that are adjusted for in the analyses you refer to.

**Reply:** We have now described the adjusting factors in the Abstract. We do not detail the specific comorbidities for which we adjusted, to limit the number of words. A sentence describing our approach to adjustment would be quite long, as adjustment for comorbidities differed by the outcome being analyzed. This can of course be found in the main text (pp8-9).

8. In the last sentence of the Abstract Methods and Findings section, please describe the main limitation(s) of the study's methodology.

**Reply:** The main limitation of the study’s methodology now has now been described at the end of the Methods and Findings section of the Abstract: “The main limitation of this study was the risk of misclassification of migraine, which could lead to underestimation of the impact of migraine on each outcome.”

9. Abstract Conclusions:

Please interpret the study based on the results presented in the abstract, emphasizing what is new without overstating your conclusions. The phrase "In this study, we observed ..." may be useful.

Please avoid vague statements such as "these results have major implications for policy/clinical care". Mention only specific implications substantiated by the results.

Please avoid assertions of primacy ("We report for the first time....")

**Reply:** We have amended the first part of the Abstract Conclusions using the suggested phrase to emphasize the conclusions applicable to our study (p3).

10. AUTHOR SUMMARY

At this stage, we ask that you include a short, non-technical Author Summary of your research to make findings accessible to a wide audience that includes both scientists and non-scientists. The Author Summary should immediately follow the Abstract in your revised manuscript. This text is subject to editorial change and should be distinct from the scientific abstract. It may be helpful to you to review some examples in papers on our website here: <https://journals.plos.org/plosmedicine/>

Please see our author guidelines for more information, here: <https://journals.plos.org/plosmedicine/s/revising-your-manuscript#loc-author-summary>

**Reply**: The following Author Summary now has been added after the Abstract (p3):

Why was this study done?

- Migraine is associated with an increased risk of myocardial infarction and stroke.
- Previous studies show that the risk of ischemic stroke among individuals with migraine is increased mainly among young women.
- Whether this is also the case for myocardial infarction and hemorrhagic stroke is unknown.

What did the researchers do and find?

- The researchers used Danish national registries to identify women and men with and without migraine living in Denmark during the years 1996–2018.
- The risk of having a myocardial infarction or stroke at age 60 or younger was examined for individuals with and without migraine, separately for men and women.
- Migraine was associated with similarly increased risk of ischemic stroke for men and women aged 60 or younger. Migraine may be associated with increased risk of myocardial infarction and hemorrhagic stroke only in women.

What do these findings mean?

- Unlike previous studies, the researchers found that the risk of ischemic stroke in individuals with migraine does not appear to differ substantially between men and women. Migraine may be associated with an increased risk of myocardial infarction and hemorrhagic stroke only among women. However, absolute risks were low.
- Given that migraine is much more prevalent in women, presence of migraine likely represents a greater societal migraine-associated disease burden among women.

11. INTRODUCTION

The introduction is rather brief and lacks some degree of context. Please expand details of the background and importance of the study. See reviewer #2 comments (below) also.

Please also indicate whether your study is novel and how you determined that.

**Reply:** We thank the Editors and Reviewer #2 for these comments. We now have elaborated on the background of our study (p4). The literature is limited regarding the question whether the impact of migraine on risk of premature myocardial infarction (MI) and hemorrhagic stroke differs according to sex. As we mentioned in the Introduction and Discussion, more studies have been conducted on the impact of migraine on risk of ischemic stroke. However, a recent review by Tietjen and Maly suggests that studies examining the impact of migraine on ischemic stroke among young men may have been underpowered [1]. This motivated our study.

We used free text and Medical Subject Headings in MEDLINE (PubMed) searches of existing literature. Although the amount of literature reporting sex-specific estimates is increasing, it remains a challenge to capture all relevant studies. We used Song *et al.’s* validated search tool *[2]* to identify studies that examined the association between migraine and MI and stroke separately for men and women. We also reviewed the references of studies included in meta-analyses of the association between migraine and MI and stroke (Ng *et al. [3]*, Sacco *et al. [4],* and Schürks *et al. [5]*) to identify any relevant studies that we had missed. We did identify one additional important study on the impact of migraine on risk of MI published since the last submission. It is now described in the Introduction as well as the Discussion. We added the following text to the Discussion:

*“*To the best of our knowledge, only Sternfeld *et al.*’s study has reported on the impact of migraine on risk of MI among young adults separately for men and women [6]. This study examined relative risk in two cohorts. For men with migraine under 40 years of age, the relative risk was 0.3 (95% CI [0.1, 2.4]) in one cohort and 0.6 (95% CI [0.1, 4.4]) in the other cohort. For men with migraine between 40 and 60 years of age, the relative risk was 0.8 (95% CI [0.5, 1.5]) in one cohort and 1.4 (95% CI [0.8, 2.5]) in the other cohort. Risk estimates appeared greatest for women with migraine below 40 years of age, for whom the relative risk was 1.5 (95% CI [0.7, 3.5]) in one cohort and 2.1 (95% CI [0.5, 9.5]) in the other cohort. The relative risk for women with migraine aged 40–60 years was 1.0 (95% CI [0.6, 1.7]) in one cohort and 1.6 (95% CI [1.0, 2.7]) in the other cohort. Although the impact of migraine appeared greater among women than men, the study’s estimates were imprecise [6]. While our study’s estimates had greater precision, they showed similarly that the impact of migraine on risk of MI on a relative scale may be slightly greater for women.”

12. METHODS and RESULTS

Please add the following statement, or similar, to the Methods: "This study is reported as per the Strengthening the Reporting of Observational Studies in Epidemiology (STROBE) guideline (S1 Checklist)."

**Reply:** The statement has been added as the first sentence of the Methods section (p5).

13. Did your study have a prospective protocol or analysis plan? Please state this (either way) early in the Methods section.

a) If a prospective analysis plan (from your funding proposal, IRB or other ethics committee submission, study protocol, or other planning document written before analyzing the data) was used in designing the study, please include the relevant prospectively written document with your revised manuscript as a Supporting Information file to be published alongside your study, and cite it in the Methods section. A legend for this file should be included at the end of your manuscript.

b) If no such document exists, please make sure that the Methods section transparently describes when analyses were planned, and when/why any data-driven changes to analyses took place.

c) In either case, changes in the analysis-- including those made in response to peer review comments-- should be identified as such in the Methods section of the paper, with rationale.

**Reply:** We did prepare a prospective analysis plan before initiating any analyses, as required by our department, the Department of Clinical Epidemiology at Aarhus University, Denmark. We now include the plan as a supplemental file. Within this text, we have added red comments explaining the reasons for the main changes in the original analysis plan.

14. For all observational studies, we ask authors to indicate the following in the manuscript text:
(1) the specific hypotheses you intended to test,
(2) the analytical methods by which you planned to test them,

(3) the analyses you actually performed, and

(4) when reported analyses differ from those that were planned, transparent explanations for differences that affect the reliability of the study's results. If a reported analysis was performed based on an interesting but unanticipated pattern in the data, please be clear that the analysis was data-driven.

**Reply:** We now include this information under ‘statistical analyses’ within the Methods section: *“*The original purpose of this study, as stated in the pre-specified protocol, was to examine the interaction between sex and migraine on the risk of premature MI and stroke. However, an interaction analysis demands two exposures that are or are not present (with one group having none, two groups having either, and one group having both). It was not clear to us whether men or women would be the ‘exposed’ sex. We therefore changed the aim of the study to examine the impact of migraine on the risk of MI and stroke among women and men separately. The reason for this change was that earlier studies revealed clear sex differences for the risk of ischemic stroke, but did not examine sex differences in the risk of MI. We therefore focused on whether the sex differences associated with ischemic stroke also applied to myocardial infarction and hemorrhagic stroke.”

15. Where you report 95% CIs please also report p values. Please report p as p<0.001 and where higher as p=0.002, for example As above, suggest reporting statistical information as follows: “…0.3% (95% CI [-0.1%, 0.6%]; p<0.001 or p=0.002), for clarity and accessibility. Please check and amend throughout for consistency in data reporting.

Please see methodological reviewer (#3) comments below which we agree with

**Reply:** We now report p-values as suggested, both in the text and in Tables 3–4.

16. TABLES

Please include p values where relevant, reported as detailed above.

In the table caption please detail the statistical test used to determine them. There is some inconsistency in how your separate upper and lower confidence limits (semicolons Vs commas). Suggest the use of commas. Please amend throughout where relevant.

**Reply:** We have added the p-values to the tables and reported the statistical tests we used to determine them. We now use commas throughout to separate lower and upper confidence limits. We thank the Editors for noticing this inconsistency.

17. FIGURES

Please consider using a colour palate which is accessible to those with colour blindness Figure 1 – does the shadow behind the lines have a meaning/purpose? Please clarify/revise as necessary.

**Reply:** We now have chosen colors that should be clear to persons with colour blindiness for Figure 1, as well as for all the figures in the Appendix. The shadowed area indicates the 95% confidence interval. This explanation has been added to the figure legends.

18. SUPPORTING FILES

Appendix page 4 – suggest removing “log-log plots” above the figure caption In my version the legends which depict blue and red lines are very small and not easy to read, please revise As above, suggest the use of commas to separate upper and lower confidence limits. Please amend throughout where relevant

**Reply:** We thank the Editors for these suggestions. We have made the legends larger and also changed the colors to make them understandable to people with difficulties in discerning certain colors. Finally, we have separated the estimates in the confidence intervals using commas throughout the Supplemental Material.

19. REFERENCES

For in-text reference call-outs, citations should be placed in square brackets and preceding punctuation as for example “…[1-4,7], or [1,5,9].”

In the bibliography please ensure that up to but no more than 6 author names are listed followed by et al., in the event that more than 6 authors contribute to the study.

Please ensure that journal name abbreviations should be those found in the National Center for Biotechnology Information (NCBI) databases.

Please see our website for other reference guidelines <https://journals.plos.org/plosmedicine/s/submission-guidelines#loc-references>

**Reply:** We have amended the in-text reference call-outs as suggested and used the requested reference style, which we downloaded via the journal’s webpage (PLoS).

## Comments from the reviewers:

**Reviewer #1:** This is an interest study investigating the association between impact of migraine on risk of premature myocardial infarction and stroke among men and women in Denmark. There are several strengths of the study: It is built on the Danish registries with diagnoses based on the National Patient Register, it is a nested-case control study with cases matched 1:5 with a random sample of the general Danish population, confounder adjustment is built on DAGS, there are appropriate sensitivity analyses, and the results are presented with both relative and absolute risks.

However, the following should be considered by the authors before publication:

20. Sex differences. In your aim you talk about men and women, corresponding to stratified analyses, but you draw conclusions regarding sex differences in the associations; however, these differences are not tested. So, if you want to examine sex differences please state p-values for the interactions between sex and migraine in all models. Otherwise, you can talk about men and women in your results and conclusions but not about sex differences.

**Reply:** We thank the Reviewer for the positive feedback on our study. Regarding sex differences, our study was motivated by previous literature indicating a difference between men and women regarding the impact of migraine on ischemic stroke. As we did not find such a difference, we argue that the impacts are similar for men and women. However, similarities cannot be proven by a p-value. For this reason, the ‘non-inferiority design’ was invented for randomized trials to circumvent the applicability of a p-value for differences. The ‘non-inferiority design’ aims to show that a given difference is, or is not, larger than a pre-specified difference. Apart from general discussion about the soundness of this design, it would seem contrived to turn the analysis of a difference in the incidence of diseases between men and women into a non-inferiority analysis--because we would have to define the non-inferiority margin ‘after the fact’. In our paper, we refer to previous studies that found rather large differences between men and women, while we did not. However, one can never be certain about the existence of smaller differences (that also may not be of great clinical significance).

21. Study design: This is a nested case-control study - it should be stated in the text under study cohorts and in the abstract.

**Reply:** We conducted a cohort study. We identified individuals with migraine using registry data. For each person with migraine we sampled five migraine-free persons from the general population in Denmark, matched on age, sex, and index year. Again using registry data, we followed all individuals until they had a myocardial infarction, stroke, turned 61 years old, emigrated, died, or our study ended. In our revised manuscript, we have attempted to make this clear by adding a graphical depiction of the study design, including windows for assessment of exposure, covariates, and exclusion, using the template provided by Schneeweiss *et al.*[7] The figure is provided in the Appendix S2.

22. Is birth equal to birthdate? (Page 4 under matching).

**Reply:** We apologize for the confusion. The sentence has been revised to read “…matched on sex, birth year, and calendar year” (p6).

23. Stroke: You separate the two types of strokes - ischemic stroke and hemorrhagic stroke - however, your results would be easier to understand if you present the two types together in the main analyses and then show the separate models after that. You also need to explain for the reader why you also show the analyses separated by stroke type if you prefer to demonstrate this as well.

**Reply:** We thank the Reviewer for this feedback. We separated the two outcomes because of the different pathogenesis of ischemic and hemorrhagic stroke. In addition, the baseline risk of ischemic stroke differed for men and women, whereas for hemorrhagic stroke the baseline risk was similar for men and women. This affects the impact migraine may have on the outcome when the impact is assessed on the relative scale. Finally, existing literature showing the risk of stroke among young men and women has mainly examined ischemic stroke. Thereby, separate estimates for ischemic and hemorrhagic stroke are desirable for comparisons with previous research. For these reasons, we performed the suggested analysis but chose not to include it in the manuscript. Of course, should the Editors find it relevant to include it in the manuscript or appendix we are happy to do so. The results of this analysis are shown below in Table 1. The results were similar to those of the main analysis in which we estimated the impact of migraine on the risk of premature ischemic stroke. We found that the impact of migraine on the risk of combined stroke was slightly attenuated for men. This was expected since we found no impact of migraine on men’s risk of premature hemorrhagic stroke in the main analyses. Finally, as suggested by the Reviewer, we have added a brief explanation of why we chose to separate the outcomes (Methods p6).

| Table 1. Absolute risk, risk difference (RD), crude and adjusted hazard ratios (HRs) for 1-20 years of follow-up for premature stroke (combined ischemic and hemorrhagic stroke). Migraine identified by prescription data. P values reflect likelihood ratio tests for HRs. | | | | | | |
| --- | --- | --- | --- | --- | --- | --- |
|  | **Absolute risk**  **% (95% CI)** | **RD within sex**  **% (95% CI)** | **Crude HR**  **(95% CI)** | **Crude HR within sex**  **(95% CI)** | **Adjusted* HR**  **(95% CI)** | **Adjusted* HR within sex**  **(95% CI)** |
| Women without migraine | 1.7 (1.6, 1.7) | - | 1.00 (Ref) | 1.00 (Ref) | 1.00 (Ref) | 1.00 (Ref) |
| Women with migraine | 2.1 (1.9, 2.2) | 0.4 (0.2, 0.5) | 1.20 (1.13, 1.27); p<0.001 | 1.20 (1.13, 1.27); p<0.001 | 1.19 (1.12, 1.26); p<0.001 | 1.19 (1.12, 1.26); p<0.001 |
| Men without migraine | 2.5 (2.4, 2.7) | - | 1.45 (1.38, 1.53); p<0.001 | 1.00 (Ref) | 1.44 (1.38, 1.52); p<0.001 | 1.00 (Ref) |
| Men with migraine | 2.9 (2.5, 3.2) | 0.4 (0.0, 0.7) | 1.68 (1.52, 1.84); p<0.001 | 1.15 (1.04, 1.28); p=0.006 | 1.62 (1.47, 1.78); p<0.001 | 1.13 (1.02, 1.25); p=0.019 |
| * Adjusted for age, calendar period, hypertension, thyroid disease, hyperlipidemia, VTE, obesity, alcohol-related disease, COPD, and atrial fibrillation/flutter. | | | | | | |

24. Cox proportional hazards assumptions: You have problems in many of your models with the Cox proportional hazards assumptions. Another way to deal with this is besides excluding the first year of follow up like you did, is to make a stratified piecewise Cox regression, so you investigate different time-periods separately. Particularly in the models where you repeat the analyses using first-time ICD-codes recorded in the DNPR to identify migraine in stead of the prescription date you run into problems - this is an important analysis. Could you fix the problem by a stratified piecewise cox regression and try to combine the two types of stroke?

**Reply:** As the Reviewer notes, we chose to compute 1-20 year risk estimates because proportionality of the hazards did not prevail for the entire follow-up period, *i.e.,* for some outcomes, the hazard functions crossed within the first year. We now have computed the risk estimates for 0-1 year of follow-up, which are shown in the table below. As the absolute risks were quite low, we chose to add a second decimal to the absolute risks and risk differences in this table. The limited number of events within the first year of follow-up resulted in imprecise results. The impact of migraine within this year appeared greater for both men and women for risk of both ischemic and hemorrhagic stroke. This raised the concern that these estimates were subject to detection bias. For this reason, as well as the lack of proportionality, we chose not to include the table in the manuscript. However, we are of course willing to add the table and amend the manuscript accordingly if required. We will leave the decision to the Editor.

| Table 2. Absolute risk, risk difference (RD), crude hazard ratios (HRs) and adjusted HRs for 0-1 years of follow-up for premature myocardial infarction, ischemic stroke, and hemorrhagic stroke. P values reflect likelihood ratio tests for HRs. | | | | | | | | |
| --- | --- | --- | --- | --- | --- | --- | --- | --- |
|  | **Absolute risk**  **% (95% CI)** | | **RD within sex**  **% (95% CI)** | **Crude HR**  **(95% CI)** | **Crude HR within sex**  **(95% CI)** | **Adjusted* HR**  **(95% CI)** | **Adjusted* HR within sex**  **(95% CI)** |  |
| Myocardial infarction |  |  |  |  |  |  |  |  |
| Women without migraine | 0.03 (0.03, 0.04) | | - | 1.00 (Ref) | 1.00 (Ref) | 1.00 (Ref) | 1.00 (Ref) |  |
| Women with migraine | 0.04 (0.03, 0.05) | | 0.01 (0.00, 0.02) | 1.18 (0.91, 1.53); p=0.22 | 1.18 (0.91, 1.53); p=0.22 | 1.12 (0.88, 1.48); p=0.327 | 1.12 (0.88, 1.48); p=0.327 |  |
| Men without migraine | 0.10 (0.08, 0.11) | | - | 2.90 (2.41, 3.48); p<0.001 | 1.00 (Ref) | 3.01 (2.51, 3.63); p<0.001 | 1.00 (Ref) |  |
| Men with migraine | 0.13 (0.09, 0.16) | | 0.03 (−0.01, 0.07) | 2.78 (2.80, 5.11); p<0.001 | 1.30 (0.96, 1.78);  p=0.094 | 3.72 (2.75, 5.04); p<0.001 | 1.28 (0.94, 1.75); p=0.119 |  |
| Ischemic stroke |  |  |  |  |  |  |  |  |
| Women without migraine | 0.03 (0.03, 0.04) | | - | 1.00 (Ref) | 1.00 (Ref) | 1.00 (Ref) | 1.00 (Ref) |  |
| Women with migraine | 0.05 (0.04, 0.06) | | 0.02 (0.01, 0.03) | 1.69 (1,34, 2.13); p<0.001 | 1.69 (1,34, 2.13); p<0.001 | 1.63 (1.29, 2.06); p<0.001 | 1.63 (1.29, 2.06); p<0.001 |  |
| Men without migraine | 0.05 (0.04, 0.06) | | - | 1.48 (1.17, 1.87); p<0.001 | 1.00 (Ref) | 1.46 (1.15, 1.85); p=0.002 | 1.00 (Ref) |  |
| Men with migraine | 0.08 (0.05, 0.11) | | 0.03 (0.00, 0.06) | 2.45 (1.69, 3.54); p<0.001 | 1.65 (1.10, 2.48); p=0.015 | 2.23 (1.53, 3.24); p<0.001 | 1.58 (1.05, 2.37); p=0.028 |  |
| Hemorrhagic stroke |  |  |  |  |  |  |  |  |
| Women without migraine | 0.02 (0.02, 0.02) | | - | 1.00 (Ref) | 1.00 (Ref) | 1.00 (Ref) | 1.00 (Ref) |  |
| Women with migraine | 0.03 (0.02, 0.04) | | 0.01 (0.01, 0.04) | 1.54 (1.15, 2.07); p=0.004 | 1.54 (1.15, 2.07); p=0.004 | 1.52 (1.13, 2.04); p=0.005 | 1.52 (1.13, 2.04); p=0.005 |  |
| Men without migraine | 0.02 (0.01, 0.02) | | - | 0.87 (0.61, 1.24); p=0.442 | 1.00 (Ref) | 0.86 (0.60; 1.22); p=0.400 | 1.00 (Ref) |  |
| Men with migraine | 0.04 (0.02, 0.06) | | 0.02 (0.00, 0.04) | 1.89 (1.13, 3.14); p=0.015 | 2.17 (1.20, 3.89); p=0.010 | 1.79 (1.07, 2.99); p=0.026 | 2.14 (1.19, 3.85); p=0.011 |  |
| *For MI: adjusted for age, calendar period, hypertension, thyroid disease, hyperlipidemia, VTE, obesity, alcohol-related disease, and COPD.  For ischemic stroke: adjusted for age, calendar period, hypertension, thyroid disease, hyperlipidemia, VTE, obesity, alcohol-related disease, COPD and atrial fibrillation/flutter.  For hemorrhagic stroke: adjusted for age, calendar period, hypertension, alcohol-related disease, COPD, and anticoagulant treatment. | | | | | | | |  |

25. Conclusion: Your conclusion should be rewritten. "A slightly increased risk (if any)" is not specific enough in the conclusion. The last part from "despite small differences…" should be moved for instance into the introduction. Your conclusion should sum up clear and concise what you find in this study, and I think a combination of the two types of stroke would make it easier.

**Reply:** We have removed the phrase, “despite small differences”, from the conclusion. Regarding being specific, we are not entirely sure what the Reviewer means. We have amended the conclusion in line with the last comment by Reviewer #3 and hope this also addresses these issues. Our response to Reviewer #3 was “ We agree… that since the risk differences were unadjusted, they should not constitute the basis for conclusions. We therefore have amended the conclusions reported in the Discussion as well as in the Abstract. Our point was also that the impact of migraine on the HRs should be seen in light of the absolute risks. As we mention, the larger baseline risks for men for MI and ischemic stroke influence the size of the impact of migraine on the relative scale.

**Reviewer #2: Review:**

It was interesting to read this study on migraine and risk for myocardial infarction, ischemic and hemorrhagic stroke. It uses large registers both use of migraine medicines and diagnoses from healthcare in a clever way. I believe the method is sound and the results are reasonable.

1. My greatest concern is labeling and the fact that almost 180 000 women redeemed migraine medicine but only approx. 25000 women received a migraine diagnose from healthcare.

**Reply:** We thank the Reviewer for the positive remarks about our study. We understand the concern about the disparity between the number of redeemed prescriptions *vs.* diagnoses. In Denmark, general practitioners generally treat migraine. In cases of severe migraine or diagnostic uncertainty, patients may be referred to the hospital. It is important to note that the Danish Patient Registry contains only hospital-based discharge diagnoses, thus lacking diagnosis codes assigned by general practitioners. This referral system explains the relatively few individuals identified with migraine based on diagnostic codes from the Danish Patient Registry. For this reason, in the main analysis we chose to identify persons with migraine using redeemed prescriptions for migraine-specific medications. Data are available on all redeemed prescriptions issued by general practitioners, specialists in private practice, and hospitals (except for treatments administered only during admissions) for all inhabitants of Denmark since 1995. Our use of migraine-specific medications to identify persons with migraine was based on the assumption that prescription data would identify more individuals with migraine than would be ascertainable from hospital registries only, and that this approach would cover all persons in the source population who were treated for migraine.

We have added this explanation to the first section of the “Study cohort” portion of the Methods section (p5).

2. I also didn't understand if a person had more than one of the three diagnoses (MI, IS or ICH) was the persons data then used several times?

**Reply:** Yes, a person could have more than one outcome diagnosis. The risks of each outcome were estimated in separate analyses to assess the entire impact of migraine on each outcome. Thus, in the analysis examining the impact of migraine on MI, we ignored whether or not a stroke occurred during follow-up and vice versa. We have clarified this approach in the statistical analyses section of the paper (see pp7-8).

3. There are also very much additional data in the appendix, consider if all are really needed (could not several pages of log-log curves be summarized in a sentence what they are showing and then "data not shown"? Are the DAG graphs necessary?).

**Reply:** We agree with the Reviewer that the Appendix contains a large amount of additional data. However, the journal guidelines suggest that summarizing data without showing them should be avoided. Moreover, the DAGs are the golden standard for illustrating the chosen adjusting variables in epidemiology. Therefore, we chose to include them. Of course, we are willing to leave them out if the Editors prefer.

4. Introduction

The main pieces are there, but the words combining them is sort of missing (like on a plate you have the main pieces of food but no sauce). Perhaps a sentence defining the knowledge gap would improve? And why is this study needed if other researchers have done similar (it is needed but argue for it)? I am not fond of this way of writing: "A 2018 meta-analysis…". Should the aim not state the age limit i.e., <60 years?

**Reply:** We thank the Reviewer for this feedback. We have amended the Introduction, to make the knowledge gap clearer. We also now state the age limit in the aim.

5. Method. How old was the youngest participant? Even if it is very rare, were there any individuals below 18 years?

**Reply:** As we only included adults, youngest study participant was 18 years old.

6. I enjoyed the use of covariates. However, I was thinking why beta-blockers are used (page 5). It seems as if it was used to identify cardiac disease or hypertension, but it is also sometimes used as prophylactic for migraine.

**Reply:** We are glad that the Reviewer appreciated the use of covariates. Indeed, beta-blockers may be used for various conditions including migraine prophylaxis. We therefore included this variable as a descriptive measure of medication use in the study population.

7. Page 6 states index-date but does not define this. Is it for the second redeemed prescription of migraine medicine or the first cardiovascular event (MI, IS or ICH)?

**Reply:** The Index date was the date on which the second prescription for migraine medication was redeemed by persons with migraine. Individuals without migraine were assigned the index date of the person with migraine to whom they were matched. The index date for individuals with migraine is defined on page 6. Information on the index date for individuals without migraine was missing from the original manuscript and now has been added to page 6.

8. During the study period there was a change in use of migraine medicine, at least an increase of triptane use. Did that affect the results?

**Reply:** The Reviewer raises an interesting point that we had not addressed. To follow up, we conducted a third sensitivity analysis in which the results were stratified by index year into two periods: 1996–2006 and 2007–2018. To allow for the same duration of follow-up in both strata, we only looked at 1–10-year risk estimates. The results are provided in Appendix S9. In brief, the impact of migraine on risk of myocardial infarction for women was similar, although slightly attenuated, in both time periods. For men an association with MI was observed only in the more recent time period (2007–2018). HRs of ischemic stroke were similar to those of the main analyses for both men and women in the two time periods. Regarding the impact of migraine on hemorrhagic stroke, the results were rather imprecise. There may have been an impact for women within the first time period, but this remains uncertain.

We discuss this sensitivity analysis in the Methods and Results section of the revised manuscript. We thank the Reviewer for suggesting this interesting analysis.

9. Results. Page 8, sixth line should not the second "4" have a decimal? (Interquartile range: 4.3-4; 14.0 years)

**Reply:** We now have corrected this typo.

10. Discussion. First paragraph hardly does not mention ischemic stroke.

**Reply:** We thank the Reviewer for pointing out this lack of information. The first line now focuses on premature ischemic stroke.

11. Table/figures. Why is not Table 1a and 1b separated? They are clearly two different Tables?

**Reply:** The tables describe the baseline characteristics of the study cohorts and were originally one table. It was split into two tables due to size. However, we agree with the Reviewer that the numbering of the tables was confusing. Therefore, we have changed the numbering from Table 1a to Table 1 and Table 1b to Table 2).

12. Table 1a the heading does not describe how migraine is defined

**Reply:** We have now added this information to the heading of the table.

13. Table 1b what is "Platelet inhibitor" and why is it separated from Clopidogrel and Aspirin? In Table A2 there is a different set up.

**Reply:** We apologize for the confusion. We separated out clopidogrel because it is one of the most commonly used prophylactic treatments for arterial thrombosis. We separated aspirin from the other platelet inhibitors, because it can be used both prophylactically and as a painkiller. However, as the Reviewer points out, this may be confusing to the reader. We therefore now report all platelet inhibitors as one variable “Platelet inhibitors”, including clopidogrel and low-dose aspirin. As high-dose aspirin (500 mg) is used as a painkiller and in many cases would be taken due to migraine, it is not included in this category.

This revision necessitated a slight change in the adjusting variable ‘anticoagulant treatment’, which now includes vitamin K antagonists, direct oral anticoagulants, and the new version of platelet inhibitors. We have amended Table 1 and the descriptions of these variables on pages 7–9 and changed the codes in Appendix S6 Table 2 accordingly.

14. Figure 1 - the grey area of the curves is not defined.

**Reply:** These areas represent 95% confidence intervals. We have now added this explanation to the figure legend.

15. Figure A3 - Why different colors? Does that have a meaning, if so clarify.

**Reply:** We assume the Reviewer refers to the different colors of the arrows and nodes; we now have added a legend explaining the colors. We also realize that the DAG may have been somewhat oversimplified. As shown in Table 1, we found that hypertension, hyperlipidemia, venous thromboembolism, thyroid disease, and obesity all were more prevalent among individuals with migraine. We therefore chose to adjust for these diseases/factors. As now illustrated by the DAG, we don’t believe that these diseases directly increase risk of migraine but rather that the factors are associated with migraine through some unknown path. Previous studies suggest that the proportion of smokers is higher among individuals with migraine, but that alcohol intake is lower [8-10]. While these findings also may be due to some unknown factors, there may also be a direct impact of alcohol on migraine (*i.e*., alcohol may trigger migraine attacks). Unfortunately, we did not have information on smoking or alcohol intake, and therefore could not adjust for these directly. We therefore chose to adjust for alcohol-related disease and chronic obstructive pulmonary disease as proxies. Mood disorders and use of several medications in Table 1 were more prevalent among individuals with migraine. We could therefore have chosen to adjust for these factors. However, because we did not have the true index date of migraine onset, we believe that some of these factors could be mediators rather than potential confounders. Still, following the suggestion of Reviewer #3, we also conducted an analysis in which we adjusted for all these factors except NSAIDs and beta-blockers (which are common migraine treatments). The results of this analysis were not substantially different from those of the main analysis.

16. Table 2 and 6A is not clearly defined what separates the two.

**Reply:** We agree the distinction between the two tables was not clear. We now have provided additional information to the descriptions at the top of the tables.

17. References. Some references use full names (for instance 5-9) and other uses first names as initials. Why the differences?

Why does the first name in the list of authors have their first name initials before the last name and the rest of the authors have their first name initials after the last name?

**Reply:** We thank the Reviewer for noticing this inconsistency. We now have updated the reference style to that of PLoS to address these problems.

**Reviewer #3:** This is an interesting and well-conducted population-based study on the impact of migraine on risk of premature myocardial infarction and stroke among men and women. However, there are a few major issues needing attention especially with statistical analyses.

18. Covariates in the adjusted Cox models. It says "Covariates were chosen based on the directed acyclic graphs provided in Appendix Figures A1–3". However, this is simply neither enough nor adequate. Confounder/covariate/model selection cannot be only based on DAGs. As this is an observational study, we need to minimize the confounding effect as much as possible therefore a full and comprehensive adjustment for the models is needed. Basically all the available variables in the table 1a and 1b needed to be considered in the adjustment, plus drinking, smoking, BMI, depression and anxiety, and substance use, if possible.

**Reply:** We agree with the Reviewer that observational studies, including ours, may be susceptible to confounding and should aim to minimize it. Many of the variables included in Tables 1a and 1b (now Table 1 and Table 2) were meant to be descriptive and were not necessarily considered confounders. Using DAGs to illustrate and select potential confounders is considered a standard approach within causal inference and epidemiology [11]. This was also considered a strength of our study by the first Reviewer. Of course, some epidemiologists and statisticians want to include as many covariates as possible, aiming to additionally adjust for ‘confounding by proxy’ such as proposed by Schneeweiss *et al.* in their multi-dimensional propensity score model [12]*.* We therefore conducted an additional analysis adjusting for additional covariates, including education, income, Charlson Comorbidity Index score, diabetes, hypertension, atrial fibrillation/flutter, venous thromboembolism, Raynaud’s disease, obesity, thyroid disease, alcohol-related disease, chronic obstructive pulmonary disorder, hyperlipidemia, and ever-redeemed prescriptions for angiotensin-converting enzyme inhibitors/angiotensin II antagonists, diuretics, and anticoagulant treatments. We have added this analysis to the manuscript (current Table 4). As the Table shows, the results of this analysis were very similar to those of our original analysis. We included all variables from Table 1 and 2 (previous Table 1a and 1b) in the model except the use of beta-blockers and NSAIDs, as we believed use of these drugs could in large part be a consequence of migraine and thereby constitute mediators rather than confounders. Another potential risk of adjusting is introducing collider bias. Potential colliders should be excluded from a multidimensional propensity score [13]. Of note, the index date (date of second redemption of a prescription for migraine medication) is not the date of migraine debut. We therefore consider it likely that use of beta-blockers and NSAIDs, and potentially socioeconomic position, could be consequences of migraine. The Reviewer also suggests including information on certain lifestyle factors, such as body mass index and smoking. Unfortunately, these are not available in the Danish registries that we used in this study.

19. Competing risk. For the Cox models, as the outcomes are MI and strokes other than all cause mortality, competing risk from deaths (n=?) needs to be addressed in the models, normally by Fine-Gray models. So far, it's not done yet in the paper.

**Reply:** We initially considered running a Fine-Gray model on the data. However, as has been discussed in the literature [14], the Cox model gives the cause-specific hazard function, while the Fine and Gray model gives the sub-distribution hazard function taking death as a competing risk into account. Thus, in our study in assessing risk of myocardial infarction, the Cox model would give the hazard of myocardial infarction in individuals who had not yet had a myocardial infarction and who were still alive. The Fine and Gray model would give the hazard of myocardial infarction in individuals who had not yet had a myocardial infarction but who may have experienced the competing event, *i.e.* death.

It thus has been suggested that the Cox model is more appropriate for etiological questions while Fine-Gray models may be more appropriate for clinical predictive models and risk-scoring systems [14]. We have added this explanation to the ‘statistical analyses’ portion of the Methods section (p8). We also reasoned that within this young population the competing risk from death would be rather small. We therefore chose to examine our study question using the Cox model. Nonetheless, we reran the analyses using a Fine-Gray model. Results are presented below in Table 3, and as can be seen, these results are very similar to the ones computed by the Cox model. We therefore chose not to include the table in the manuscript or the appendix, but are of course willing to if requested.

| **Table 3. Crude and adjusted hazard ratios (HRs) of premature myocardial infarction, ischemic stroke, and hemorrhagic stroke computed using a Fine and Gray model.** | | | | |
| --- | --- | --- | --- | --- |
|  | **Crude HR**  **(95% CI)** | **Crude HR within sex (95% CI)** | **Adjusted* HR**  **(95% CI)** | **Adjusted* HR within sex**  **(95% CI)** |
| **Myocardial infarction** | | | | |
| Women without migraine | 1.00 (Ref) | 1.00 (Ref) | 1.00 (Ref) | 1.00 (Ref) |
| Women with migraine | 1.24 (1.15, 1.33); p<0.001 | 1.24 (1.15, 1.33); p<0.001 | 1.22 (1.14, 1.32); p<0.001 | 1.22 (1.14, 1.32); p<0.001 |
| Men without migraine | 3.05 (2.90, 3.21); p<0.001 | 1.00 (Ref) | 3.16 (3.00, 3.32); p<0.001 | 1.00 (Ref) |
| Men with migraine | 3.31 (3.03, 3.62); p<0.001 | 1.09 (0.99, 1.19); p=0.083 | 3.34 (3.05, 3.66); p<0.001 | 1.07 (0.98, 1.18); p=0.140 |
| **Ischemic stroke** | | | | |
| Women without migraine | 1.00 (Ref) | 1.00 (Ref) | 1.00 (Ref) | 1.00 (Ref) |
| Women with migraine | 1.23 (1.14, 1.31); p<0.001 | 1.23 (1.14, 1.31); p<0.001 | 1.22 (1.14, 1.30); p<0.001 | 1.22 (1.14, 1.30); p<0.001 |
| Men without migraine | 1.58 (1.49, 1.68); p<0.001 | 1.00 (Ref) | 1.57 (1.48, 1.67); p<0.001 | 1.00 (Ref) |
| Men with migraine | 1.98 (1.78, 2.21); p<0.001 | 1.26 (1.12, 1.41); p<0.001 | 1.91 (1.72, 2.14), p<0.001 | 1.24 (1.11, 1.39); p<0.001 |
| **Hemorrhagic stroke** | | | | |
| Women without migraine | 1.00 (Ref) | 1.00 (Ref) | 1.00 (Ref) | 1.00 (Ref) |
| Women with migraine | 1.13 (1.03, 1.24); p=0.013 | 1.13 (1.03, 1.24); p=0.013 | 1.13 (1.02, 1.24); p=0.014 | 1.13 (1.02, 1.24); p=0.014 |
| Men without migraine | 1.16 (1.06, 1.28); p=0.001 | 1.00 (Ref) | 1.14 (1.04, 1.25); p=0.005 | 1.00 (Ref) |
| Men with migraine | 1.01 (0.83, 1.23); p=0.930 | 0.87 (0.70, 1.07); p=0.185 | 0.97 (0.80, 1.19); p=0.786 | 0.85 (0.69, 1.05), p=0.137 |
| *For MI: adjusted for age, calendar period, hypertension, thyroid disease, hyperlipidemia, VTE, obesity, alcohol-related disease, and COPD. For ischemic stroke: adjusted for age, calendar period, hypertension, thyroid disease, hyperlipidemia, VTE, obesity, alcohol-related disease, COPD and atrial fibrillation/flutter. For hemorrhagic stroke: adjusted for age, calendar period, hypertension, alcohol-related disease, COPD, and anticoagulant treatment | | | | |

20. It's adequate to use the Aalen-Johansen product-limit estimator, treating death as a competing risk, for cumulative incidences of the outcomes. However, P-values are needed for the statistical differences between the curves (eg. migraine vs non-migraine). This applies to Figure 1 and all other figures in the appendix.

**Reply:** We now have used the Gray’s test to compute the p-values and have added them to the figure legends of the cumulative incidence plots.

21. Interpretation of the results. It says in the conclusion of the abstract that "Migraine was associated with similarly increased risks of premature MI and ischemic stroke among men and women and, potentially, a minor increased risk of premature hemorrhagic stroke only among women". However, only those adjusted HRs gave conclusive answers, not those AR or RD as they are not adjusted at all. For this type of cohort study, fully-adjusted analyses are absolutely essential. By looking at those adjusted HRs, we can only say migraine is associated with increased risk of MI, ischemic and hemorrhagic stroke mainly for women, not for men (except for ischemic stroke).

**Reply**: We agree with the Reviewer that since the risk differences were unadjusted, they should not constitute the basis for conclusions. We therefore have amended the conclusions reported in the Discussion as well as in the Abstract. Our point was also that the impact of migraine on the HRs should be seen in light of the absolute risks. As we mention, the larger baseline risks for men for MI and ischemic stroke influence the size of the impact of migraine on the relative scale.

**Additional notes from the authors**

During the revision process, we changed the term ‘non-vitamin K antagonist oral anticoagulants’ to ‘direct oral anticoagulants’, because the latter is the more specific term for the drugs we included in this category. We also noticed a small coding error in which a few individuals who died from their MI or stroke were coded just as dying instead of having an event. We have corrected this error in all the analyses. As can be seen from the tables, this did materially change our results. Finally, we have also changed the names of the Appendix/Supporting information according to the journal guidelines.

## References

1.	Tietjen GE, Maly EF. Migraine and Ischemic Stroke in Women. A Narrative Review. Headache. 2020;60(5):843-63. Epub 2020/04/05. doi: 10.1111/head.13796. PubMed PMID: 32246455.

2.	Song MM, Simonsen CK, Wilson JD, Jenkins MR. Development of a PubMed Based Search Tool for Identifying Sex and Gender Specific Health Literature. J Womens Health (Larchmt). 2016;25(2):181-7. Epub 2015/11/12. doi: 10.1089/jwh.2015.5217. PubMed PMID: 26555409; PubMed Central PMCID: PMCPMC4761798.

3.	Ng CYH, Tan BYQ, Teo YN, Teo YH, Syn NLX, Leow AST, et al. Myocardial infarction, stroke and cardiovascular mortality among migraine patients: a systematic review and meta-analysis. J Neurol. 2022;269(5):2346-58. Epub 2022/01/09. doi: 10.1007/s00415-021-10930-x. PubMed PMID: 34997286.

4.	Sacco S, Ornello R, Ripa P, Pistoia F, Carolei A. Migraine and hemorrhagic stroke: a meta-analysis. Stroke. 2013;44(11):3032-8. Epub 2013/10/03. doi: 10.1161/strokeaha.113.002465. PubMed PMID: 24085027.

5.	Schürks M, Rist PM, Bigal ME, Buring JE, Lipton RB, Kurth T. Migraine and cardiovascular disease: systematic review and meta-analysis. Bmj. 2009;339:b3914. Epub 2009/10/29. doi: 10.1136/bmj.b3914. PubMed PMID: 19861375; PubMed Central PMCID: PMCPMC2768778 the Deutsche Forschungsgemeinschaft and honorariums from LEK Consulting for telephone surveys. MEB is a full time employee of Merck Research Laboratories; he has carried out research, been on the advisory board, or has been in the speaker’s bureau of several pharmaceutical companies that market drugs for migraine. JEB has received investigator initiated research funding and support from the National Institutes of Health and Dow Corning; research support for pills or packaging from Bayer Heath Care and the Natural Source Vitamin E Association; and an honorarium from Bayer for speaking engagements. RBL has consulted for, carried out studies funded by, or received lecture honorariums from Merck and other companies including Allergan, GlaxoSmithKline, Johnson and Johnson, Minster, and Neuralieve. He has stock options in Minster and Neuralieve. TK has received investigator initiated research funding from the National Institutes of Health, McNeil Consumer & Specialty Pharmaceuticals, Merck, and Wyeth Consumer Healthcare; he is a consultant to i3 Drug Safety and World Health Information Science Consultants, LLC, and he has received honorariums from Genzyme, Merck, and Pfizer for educational lectures.

6.	Sternfeld B, Stang P, Sidney S. Relationship of migraine headaches to experience of chest pain and subsequent risk for myocardial infarction. Neurology. 1995;45(12):2135-42. Epub 1995/12/01. doi: 10.1212/wnl.45.12.2135. PubMed PMID: 8848181.

7.	Schneeweiss S, Rassen JA, Brown JS, Rothman KJ, Happe L, Arlett P, et al. Graphical Depiction of Longitudinal Study Designs in Health Care Databases. Ann Intern Med. 2019;170(6):398-406. Epub 2019/03/12. doi: 10.7326/m18-3079. PubMed PMID: 30856654.

8.	Scher AI, Terwindt GM, Picavet HS, Verschuren WM, Ferrari MD, Launer LJ. Cardiovascular risk factors and migraine: the GEM population-based study. Neurology. 2005;64(4):614-20. Epub 2005/02/25. doi: 10.1212/01.Wnl.0000151857.43225.49. PubMed PMID: 15728281.

9.	Winsvold BS, Hagen K, Aamodt AH, Stovner LJ, Holmen J, Zwart JA. Headache, migraine and cardiovascular risk factors: the HUNT study. Eur J Neurol. 2011;18(3):504-11. Epub 2010/09/10. doi: 10.1111/j.1468-1331.2010.03199.x. PubMed PMID: 20825470.

10.	Le H, Tfelt-Hansen P, Skytthe A, Kyvik KO, Olesen J. Association between migraine, lifestyle and socioeconomic factors: a population-based cross-sectional study. J Headache Pain. 2011;12(2):157-72. Epub 2011/03/11. doi: 10.1007/s10194-011-0321-9. PubMed PMID: 21390550; PubMed Central PMCID: PMCPMC3072515.

11.	Lash TL, Rothman KJ, VanderWeele TJ, Haneuse S. Modern Epidemiology. 4 ed: Wolters Kluwer; 2020.

12.	Schneeweiss S, Rassen JA, Glynn RJ, Avorn J, Mogun H, Brookhart MA. High-dimensional propensity score adjustment in studies of treatment effects using health care claims data. Epidemiology. 2009;20(4):512-22. Epub 2009/06/03. doi: 10.1097/EDE.0b013e3181a663cc. PubMed PMID: 19487948; PubMed Central PMCID: PMCPMC3077219.

13.	Rassen JA, Blin P, Kloss S, Neugebauer RS, Platt RW, Pottegård A, et al. High-dimensional propensity scores for empirical covariate selection in secondary database studies: Planning, implementation, and reporting. Pharmacoepidemiol Drug Saf. 2023;32(2):93-106. Epub 2022/11/10. doi: 10.1002/pds.5566. PubMed PMID: 36349471.

14.	Austin PC, Lee DS, Fine JP. Introduction to the Analysis of Survival Data in the Presence of Competing Risks. Circulation. 2016;133(6):601-9. Epub 2016/02/10. doi: 10.1161/circulationaha.115.017719. PubMed PMID: 26858290; PubMed Central PMCID: PMCPMC4741409.
