# Supplementary material for: Migraine and risk of premature myocardial infarction and stroke among men and women: A Danish population-based cohort study
Source: PLoS Med. 2023 Jun 13;20(6):e1004238. doi: 10.1371/journal.pmed.1004238 (PMC10263301; doi:10.1371/journal.pmed.1004238)
Supplement: Attachment — Submitted filename: Point-by-point_response_R2.docx [file pmed.1004238.s011.docx]

# Point-by-point response

We thank the Editorial team and Reviewers for their time, their positive feedback on the revision, and their additional comments. Below, we have addressed each comment. Page numbers refer to those in the marked version of the manuscript.

1. TITLE

Suggest “Migraine and risk of…” instead

**Reply:** We thank the Editor for this suggestion and have changed the title accordingly.

2. ABSTRACT

Line 37 – “Five times as many individuals…” onwards, would it be simpler to state “These individuals were matched 1:5 with a random sample of the general population…” or similar?

**Reply:** We have changed the wording in the abstract so this sentence now reads: “These individuals were matched on sex, index year, and birth year 1:5 with a random sample of the general population who did not use migraine-specific medication.”

3. AUTHOR SUMMARY

This largely reads very nicely. However, you state that migraine increases risk of MI and stroke (1st statement) then latterly state that the risk of MI is unknown, presumably you are referring to sex specific risk Vs total (affected) population risk? Please amend/revise for clarity.

**Reply:** We thank the Editor the positive remark on the author summary. We agree that the mentioned section was unclear. We have revised the third bullet point so it now reads: “Whether the increased risk of myocardial infarction and hemorrhagic stroke among persons with migraine also mainly pertains to women is unknown.”

4. Line 80 – sentence beginning “Migraine may be…” suggest making a separate bullet point

**Reply:** As suggested, we have made this a separate bullet point (p3).

5. TABLES

Table 1 title/caption – suggest, “… [n, (%)]…”

**Reply:** As suggested, we have changed this in the caption of Table 1 and 2.

6. REFERENCES

For in-text reference callouts, please remove spaces between different citations. For example, line 430 should read “…[16,31]…” as opposed to “…[16, 31]…” please check and amend throughout.

**Reply:** We have changed the in-text callouts as suggested.

Comments from Reviewers:

Reviewer #1: The authors have addressed all my comments from the first version of the manuscript or at least answered why they have not changed the manuscript accordingly.

I can accept the current version and will let the editor decide whether the extra tables should be included in the manuscript.

Reviewer #2: I think the authors have done an excellent job answering my concerns! Even though my epidemiological skills are not at a level to fully understand the concerns mentioned by Reviewer #3, I feel that I am convinced by the results of the paper.

Only minor things, optional for the authors to change:

7. Line 391-408. It is interesting to discus the possible ways that migraine can cause MI och stroke, but are there theories about the sex difference? Sex is only discussed concerning antithrombotics, not focused on the main topic of the paper.

**Reply:** We thank the Reviewer for this very relevant suggestion. We have added the following to this part of the discussion: “Due to the preponderance of women among persons with migraine and the link between frequency of migraine attacks and hormone fluctuations during the menstrual cycle, estrogen is believed to be involved in migraine pathophysiology [1]. Furthermore, endogenous estrogen is believed to be protective of cardiovascular disease [2]. Thereby, estrogen has been suggested to be the link between migraine and cardiovascular risk among women [3]. However, as our findings suggest the increased risk of at least ischemic stroke also exists among young men, other mechanisms may be at play.”

8. In "S8 - Sensitivity analysis 2: Identifying migraine using diagnosis codes in the DNPR." the figure for women and men with/without migraine are all straight lines. If printed on black and white it would be easier if some lines are straight, others dotted, lined and so on.

**Reply:** We thank the reviewer for also considering the graphical layout. However, as this figure will only be part of the online supporting information, we have chosen to keep its current layout.

Reviewer #3: Many thanks authors for their great effort to improve the manuscript. All my comments/concerns were comprehensively addressed. I am satisfied with the response and revision. No further issues needing atttention.

## References

1.	Allais G, Chiarle G, Sinigaglia S, Airola G, Schiapparelli P, Benedetto C. Estrogen, migraine, and vascular risk. Neurol Sci. 2018;39(Suppl 1):11-20. Epub 2018/06/16. doi: 10.1007/s10072-018-3333-2. PubMed PMID: 29904828.

2.	Iorga A, Cunningham CM, Moazeni S, Ruffenach G, Umar S, Eghbali M. The protective role of estrogen and estrogen receptors in cardiovascular disease and the controversial use of estrogen therapy. Biol Sex Differ. 2017;8(1):33. Epub 2017/10/27. doi: 10.1186/s13293-017-0152-8. PubMed PMID: 29065927; PubMed Central PMCID: PMCPMC5655818.

3.	Linstra KM, Ibrahimi K, Terwindt GM, Wermer MJ, MaassenVanDenBrink A. Migraine and cardiovascular disease in women. Maturitas. 2017;97:28-31. Epub 2017/02/06. doi: 10.1016/j.maturitas.2016.12.008. PubMed PMID: 28159058.
